# Rational Design of Chitin Deacetylase Inhibitors for Sustainable Agricultural Use Based on Molecular Topology

**DOI:** 10.1021/acs.jafc.2c02377

**Published:** 2022-10-04

**Authors:** Riccardo Zanni, Jesús Martínez-Cruz, María Gálvez-Llompart, Dolores Fernández-Ortuño, Diego Romero, Ramón García-Domènech, Alejandro Pérez-García, Jorge Gálvez

**Affiliations:** † Molecular Topology and Drug Design Unit, Department of Physical Chemistry, University of Valencia, 46010Valencia, Spain; ‡ Departamento de Microbiología, Facultad de Ciencias, Universidad de Málaga, Málaga29071, Spain; § Instituto de Hortofruticultura Subtropical y Mediterránea “La Mayora”, Consejo Superior de Investigaciones Científicas (IHSM-UMA-CSIC), Universidad de Málaga, Málaga29071, Spain

**Keywords:** crop protection, fungicide resistance, molecular topology, QSAR, pest control, powdery mildew, pesticide design

## Abstract

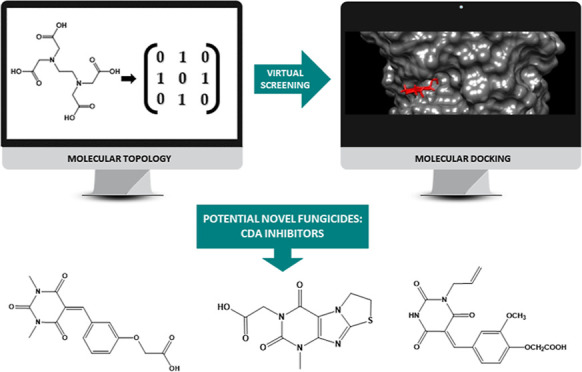

Fungicide resistance is a major concern in modern agriculture; therefore, there is a pressing demand to develop new, greener chemicals. Chitin is a major component of the fungal cell wall and a well-known elicitor of plant immunity. To overcome chitin recognition, fungal pathogens developed different strategies, with chitin deacetylase (CDA) activity being the most conserved. This enzyme is responsible for hydrolyzing the *N*-acetamido group in *N*-acetylglucosamine units of chitin to convert it to chitosan, a compound that can no longer be recognized by the plant. In previous works, we observed that treatments with CDA inhibitors, such as carboxylic acids, reduced the symptoms of cucurbit powdery mildew and induced rapid activation of chitin-triggered immunity, indicating that CDA could be an interesting target for fungicide development. In this work, we developed an *in silico* strategy based on QSAR (quantitative structure-activity relationship) and molecular topology (MT) to discover new, specific, and potent CAD inhibitors. Starting with the chemical structures of few carboxylic acids, with and without disease control activity, three predictive equations based on the MT paradigm were developed to identify a group of potential molecules. Their fungicidal activity was experimentally tested, and their specificity as CDA inhibitors was studied for the three best candidates by molecular docking simulations. To our knowledge, this is the first time that MT has been used for the identification of potential CDA inhibitors to be used against resistant powdery mildew strains. In this sense, we consider of special interest the discovery of molecules capable of stimulating the immune system of plants by triggering a defensive response against fungal species that are highly resistant to fungicides such as powdery mildew.

## 1 Introduction

To achieve the production of high-quality crops with optimal yields, growers have to protect them from damage by different pests. The use of pesticides has become an integral part of modern agriculture; however, the use of chemicals suffers from increasing problems of resistance in the target organisms. For example, the powdery mildew fungi (*Erysiphales*) are notorious as “high-risk” organisms for rapid resistance development.^1^ Powdery mildew infects nearly 10,000 species of angiosperms, including economically important crops such as cereals, grapes, and many vegetables and ornamental plants.^2^ As disease management is highly dependent on chemicals, cases of fungicide resistance have been widely reported in these fungi. For example, fungicide resistance in the cucurbit powdery mildew pathogen *Podosphaera xanthii* is a major problem in southern Spain, with multi-resistant isolates found in areas of more intense cropping.^3−7^ Today, fungicide-based plant protection is indispensable for efficient and large-scale crop production. New fungicides are urgently needed to meet this challenge.

Fungal cell walls are dynamic structures that are essential for cell viability, morphogenesis, and pathogenesis, and they are the first defense barrier against fungal pathogens.^8^ An important structural component of fungal cell walls and a well-known elicitor of immune response in plants is chitin, a long-chain polymer of β-1,4-*N*-acetylglucosamine, a derivative of glucose.^9^ As a consequence of plant enzymatic activities, small chitin oligomers are released that can be recognized by plant receptors, promoting the activation of the so-called chitin-triggered immunity.^10^ To counter this response, fungal pathogens have evolved strategies to manipulate chitin detection, such as the secretion of effector proteins that sequester or degrade immunogenic chitin oligomers,^11,12^ thus avoiding their recognition by the plant. Another mechanism involved in disarming chitin-triggered immunity is the activity of the chitin deacetylase (CDA) enzyme. CDA is a widely conserved enzyme in fungi that catalyzes the hydrolysis of the *N*-acetamido group in *N*-acetylglucosamine units of chitin oligomers, promoting its conversion to chitosan, the deacetylated chitin derivative, which can no longer bind to chitin receptors.^13,14^

When it comes to designing and discovering new chemicals with specific biological activity, one of the most promising computer-aided drug design methods is molecular topology (MT) combined with QSAR (quantitative structure-activity relationship). Contrary to the rest of the quantitative structure–activity relationship (QSAR) methods, the MT paradigm relies on chemo-mathematical descriptors. The methodology allows a fast and precise prediction of many biological and physicochemical properties.^15−17^ Defined as a part of mathematical chemistry, MT is related to the assimilation between molecules and graphs,^18,19^ so that it can depict molecular structures through graph theoretical indices.^20,21^ Besides, it deals with the connectivity of atoms in molecules and not with geometrical features such as angles, distances, or tridimensional structures, which are common in standard/conventional approaches.^15,22^ This way, graph theory and surrounding disciplines stand as basic tools for MT development. By this approach, excellent results have been obtained in the design and selection of new drugs for different pharmacological fields^23−25^ and more recently in crop protection.^26,27^

The fungal cell wall is a preferred and safe target for fungicides. Most of the major cell wall components and enzymes that assemble them are not present in humans, other mammals, or plants.^8^ In previous work, we identified that CDA could be a potential target for fungicide design because silencing the *P. xanthii* CDA gene or treatment of powdery mildew-infected melon cotyledons with carboxylic acids, well-known CDA inhibitors, suppressed the fungal growth in both cases as a consequence of the activation of chitin-triggered immunity.^28^ In this work, we aimed to devise an *in silico* strategy based on MT to identify new CDA inhibitors for agricultural use. Our results showed the identification of fungicidal compounds that provided disease control by activating plant chitin signaling. These CDA inhibitors are promising candidates for the development of new agricultural pesticides and, for this purpose, the patent ES20190030440-20190517 has been recently granted.^29^ Furthermore, our results are the proof of concept that demonstrates great potential for the combination of molecular biology and MT for the rational discovery of new agrochemicals.

## 2 Materials and Methods

### 2.1 Computational Methods

#### 2.1.1 Chemo-Mathematical Characterization of the Molecules

Graph theory was applied to calculate topological and topo-chemical descriptors, codifying information about the molecular structures in a purely numerical way. The 2D structures of the molecules used in this study were drawn using ChemDraw Ultra (version 10.0)^30^ and characterized by a set of different topological indexes (TIs), such as connectivity indices, topological descriptors, eigenvalue-based indices, and 2D autocorrelation descriptors.^31^ Calculation of topological and topo-chemical descriptors was performed using Dragon software.^32^

#### 2.1.2 Statistical Modeling Techniques

##### 2.1.2.1 Linear Discriminant Analysis

Linear discriminant analysis (LDA) is a method of pattern recognition capable of determining a linear combination of variables (TIs in this case) to qualitatively discriminate between two or more categories or groups of objects (molecules in our case) and enable their classification.^33^ Based on their fungicidal activity, compounds were allocated into active or inactive groups. Mahalanobis distance (distance of each case to the mean of all cases in a category) was the main classification indicator, while Wilks’ parameter λ was used to determine the robustness and value of the discriminant function. Finally, the Fischer–Snedecor parameter (*F*) was used to select the variables, following a stepwise strategy. All these parameters were calculated using STATISTICA 9.^34^ To give a clear and simple interpretation of the results obtained from the LDA analysis, the fungicidal activity distribution diagrams (FDDs) were depicted. An activity distribution diagram is a plot of expectancy of activity versus the numerical outputs of discriminant function (DF) for a particular biological activity.^35^ Expectancy of activity is defined as *E*_a_ = *a*/(*i* + 1), where “*a*” and “*i*” are, respectively, the number of active and inactive compounds in a particular interval of DF values. Similarly, we can define *E*_i_ or expectancy of inactivity as *E*_i_ = *i*/(*a* + 1). The use of such diagrams eases the visualization of DF intervals where there is a maximum probability of activity or inactivity. LDA was applied to obtain the two discriminant functions: DF_1_ and DF_2_.

##### 2.1.2.2 Multi-linear Regression Analysis

The general objective of multi-linear regression analysis (MLRA) is to define the relationship between two or more independent variables and a dependent variable by providing a linear equation to observe the data.^36^ A regression function was calculated by correlating the experimental values of fungal growth log inhibition with TIs using the software package Statistica version 9.0.^34^ The Furnival–Wilson algorithm was employed to find the best subsets of descriptors, and the selection of the regression equation was determined with minimal Mallows’ Cp parameter.^37^

#### 2.1.3 Topological Models’ Validation

Since the initial data set was very small with *n* = 10 and *n* = 8 for eqs 1 (LDA) and 2 (MLRA), respectively, internal validation or cross-validation with a leave-one-out procedure (LOO) was used to test the model’s robustness and reliability. In the LOO algorithm, one case is eliminated from the data set and then the regression and discrimination analysis, with the N-1 remaining cases and the original descriptors (the ones selected in the first regression), is performed again. The corresponding property value for the removed case is then predicted. This procedure is repeated as many times as there are cases in the data. The value of the prediction coefficient, *Q*^2^, gives insights about the quality of the prediction function selected.^38,39^ For eq 3 (DF_2_), with an *n* = 33, a “leave some out” validation method was selected. This internal validation methodology is a variant of the cross-validation method “leave-one-out” procedure where data sets were divided into subgroups: LSO1, LSO2, LSO3, and LSO4. We assigned approximately 25% of the compounds from both active and inactive for the composition of the subgroups. Next, three subgroups were used to build the LDA model, and one of the four subgroups was used as a test set.

#### 2.1.4 Molecular Docking

To confirm the results from the topological equations and the biological assays and to identify the putative binding sites of the compounds to CDA protein models, molecular docking experiments were conducted using the SwissDock online server.^40^ Docking poses were visualized using UCSF Chimera software.^41^ The 3D protein models were constructed using the crystal structures of CDA from the causal agent of common bean anthracnose *Colletotrichum lindemunthianum* (2IW0)^42^ retrieved from the Protein Data Bank (PDB). The receptor proteins and ligands were prepared by removing default ligands, water charges, and by adding polar hydrogen charges.^43^ Docking was performed by following standard procedures for a “blind docking”, that is, covering the whole surface of the protein without assigning a specific set of coordinates and “accurate” parameters. A “five best subset binding score” was calculated for the compounds used in these *in silico* experiments.^40^

### 2.2 Biological Assays

#### 2.2.1 Fungicidal Activity Tests

The fungicidal activity of the compounds identified by MT screenings was tested by four different assays: leaf disc, plant, fruit, and microplate assays.

##### 2.2.1.1 Leaf Disc Assay

For this assay, zucchini cotyledon discs and two isolates of *P. xanthii*, 2086 and SF60, were used. The assay was conducted as previously described^3^ with minor modifications. Before the application of treatments, the cotyledon discs were inoculated in the center with *P. xanthii* conidia and incubated under a 16 h light/8 h dark cycle at 25 °C for 24 h. After this incubation, the discs were immersed in the corresponding compound solution and incubated under the same conditions for 7 days. After incubation, fungal development on the leaf surface was evaluated by image analysis.^44^ For this analysis, pictures were captured with a digital camera at a fixed distance of 20 cm from the discs. The pictures were analyzed using the free Java image processing software ImageJ to calculate the surface covered by powdery mildew symptoms in each disc.

##### 2.2.1.2 Plant Assays

For plant assays, 2 week old melon seedlings or 6 week old melon plants were used.^44^ In both cases, plants were inoculated by spreading with a suspension of *P. xanthii* conidia (1 × 10^4^ conidia/mL) until the point of runoff. Twenty-four hours after inoculation, leaves were sprayed with the compound solution. Plants were then maintained in a growth chamber under a 16 h light/8 h dark cycle at 25 °C for 12 days. After this incubation, disease symptoms were evaluated by image analysis as indicated above.

##### 2.2.1.3 Fruit Assays

For fruit assays, commercial tomato and orange fruits were used and inoculated with spores of the tomato gray mold and citrus green mold agents, *Botrytis cinerea* and *Penicillium digitatum*, respectively.^45,46^ Eight hours after inoculation of the fruits with 30 μL of the corresponding spore suspension (1 × 10^3^ spores/mL), the compounds were applied by immersing the fruits in the compound solutions for 1 min in the case of tomatoes or 2 min in the case of oranges. The fruits were then incubated in boxes in the dark and at the appropriate temperature, 22 °C for tomatoes or 25 °C for oranges, for 5–6 days until the development of symptoms. Disease symptoms (fruit surface covered by fungi) were evaluated by digital analysis as indicated above.

##### 2.2.1.4 Microplate Assay

The microplate reader assay for fungal growth inhibition was done as previously described.^47^ Fungi were grown in 1 mL of PDB (potato dextrose broth) in 24-well plates, which were supplemented with the different compounds to reach final concentrations ranging from 0 to 200 μM. The wells were inoculated with 30 μL of spore suspensions of 10^4^ conidia/mL, and then, the plates were incubated for 72 h at 22 °C for *B. cinerea* or at 25 °C in the case of *P. digitatum*.

### 2.3 Enzyme Inhibition Assay

For this assay, two CDA proteins were identified in *P. xanthii*, PxCDA1 (accession number: KX495502) and PxCDA2 (accession number: KX495503),^28^ were expressed *in vitro* in *E. coli* and exposed to the selected compounds. Recombinant *N*-terminal 6-His tagged proteins were produced following standard procedures.^48^ The enzymatic activity was determined using the fluorescamine method using colloidal chitin as a substrate.^13^ To test the CDA inhibitory activity of selected compounds, the enzymatic reaction was conducted in the absence or presence of the compounds at 10 and 100 μM. The reaction mixtures were incubated for 45 min at 37 °C. After incubation, reactions were stopped with 0.4 M borate buffer (pH 9.0). After data recording, percentages of inhibition of enzyme activity were calculated.

### 2.4 Gene Silencing Experiments

The compounds with the best fungicidal potential were analyzed further to determine their mode of action as CDA inhibitors. To provide biological evidence of CDA inhibition, host-induced gene silencing assays were conducted. If the inhibition of a fungal CDA causes the activation of chitin-triggered immunity and the suppression of fungal growth, the application of CDA inhibitors to leaf tissues in which the chitin elicitor kinase receptor *CERK1* gene is silenced should have no effect on fungal growth. Silencing of melon *CmCERK1* was conducted using ATM-HIGS (*Agrobacterium tumefaciens*-mediated host-induced gene silencing) and 2 week old melon cotyledons.^48^ Twenty-four hours after agro-infiltration, plants were inoculated with a suspension of *P. xanthii* conidia (1 × 10^4^ conidia/mL), and 24 h later, the leaves were sprayed with the compound solution (100 μM). Plants were then maintained in a growth chamber under a 16 h light/8 h dark cycle at 25 °C and subsequently examined for activation of defense responses and disease symptom development. Seventy-two hours after inoculation, visualization of the oxidative burst, a defense response typical of chitin-triggered immunity, was studied by histochemical analysis of the accumulation of hydrogen peroxide (H_2_O_2_) by following the DAB-uptake method.^48^ Twelve days after inoculation, disease symptoms were evaluated by image analysis as indicated above.

## 3 Results and Discussion

### 3.1 Computational Strategy

#### 3.1.1 Computer-Aided Fungicidal Design Iterative Workflow for the Identification of Potential CAD Inhibitors

**Figure 1:**
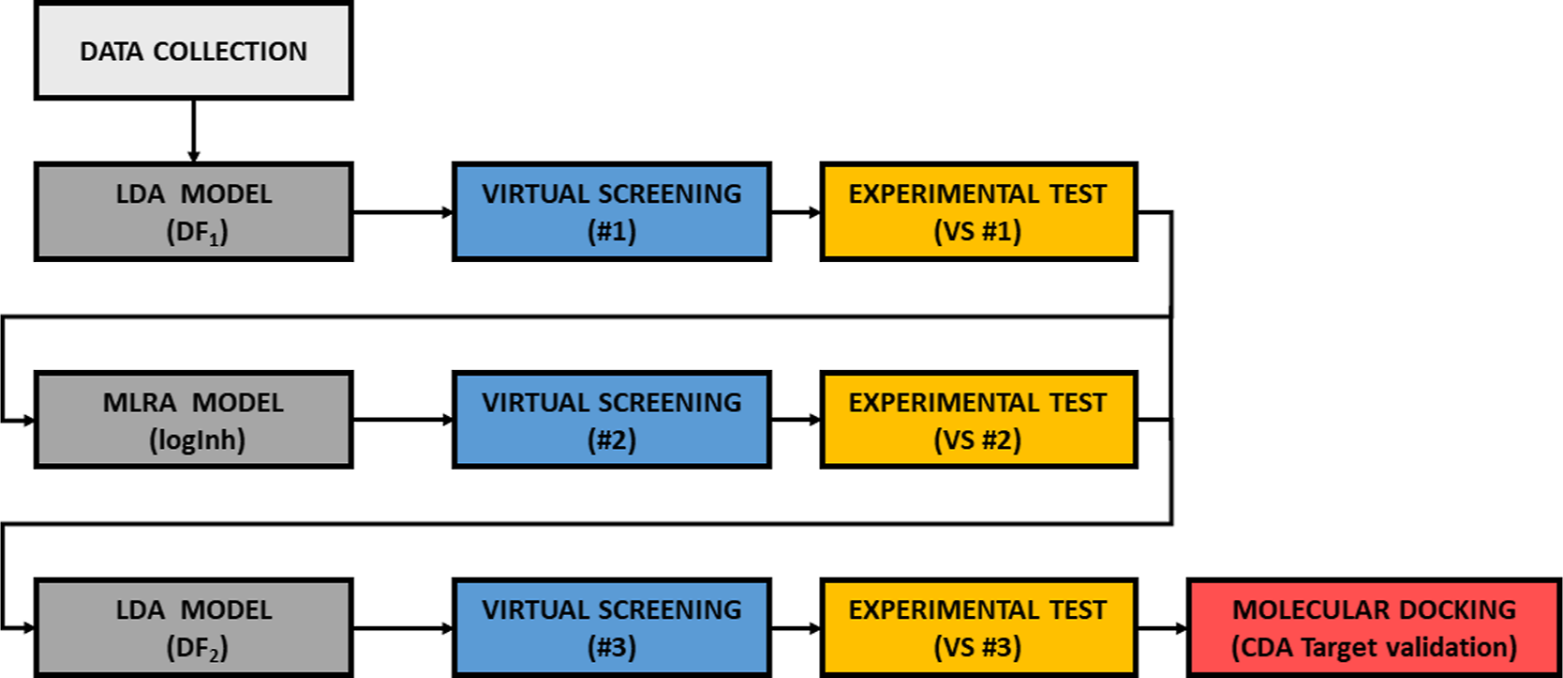
Computational strategy followed for the discovery of new fungicidal *hits* based on MT using LDA and MLRA.

A computational strategy based on MT has been developed for the identification of potential fungicides with inhibitory CAD activity, combining QSAR and molecular docking. In Figure 1, the iterative strategy followed in the present study is reported.

First, a small database of compounds with reported anti-CDA activity has been collected. In terms of CDA inhibitors, only a few carboxylic acids were described in the literature: EDTA, (GlcNAc)_2_, lactic acid, and propionic acid. These molecules were tested *in vitro*, finding insights of fungicidal activity at concentrations above 10 mM. Starting with the chemical structures of those carboxylic acid derivatives, a training set with active and inactive anti-CDA compounds was built and the first topological predictive equation (DF_1_) was developed. According to the MT approach, the chemical structure of each molecule was converted into a graph and then converted into a specific set of 2D topological descriptors applying graph theory. In Table S1, the training set of molecules used in the first discriminant model, as well as the classification results and descriptors’ values are reported. LDA was the statistical technique used to develop the first discriminant function (DF_1_)
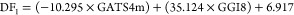
1
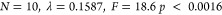


**Figure 2:**
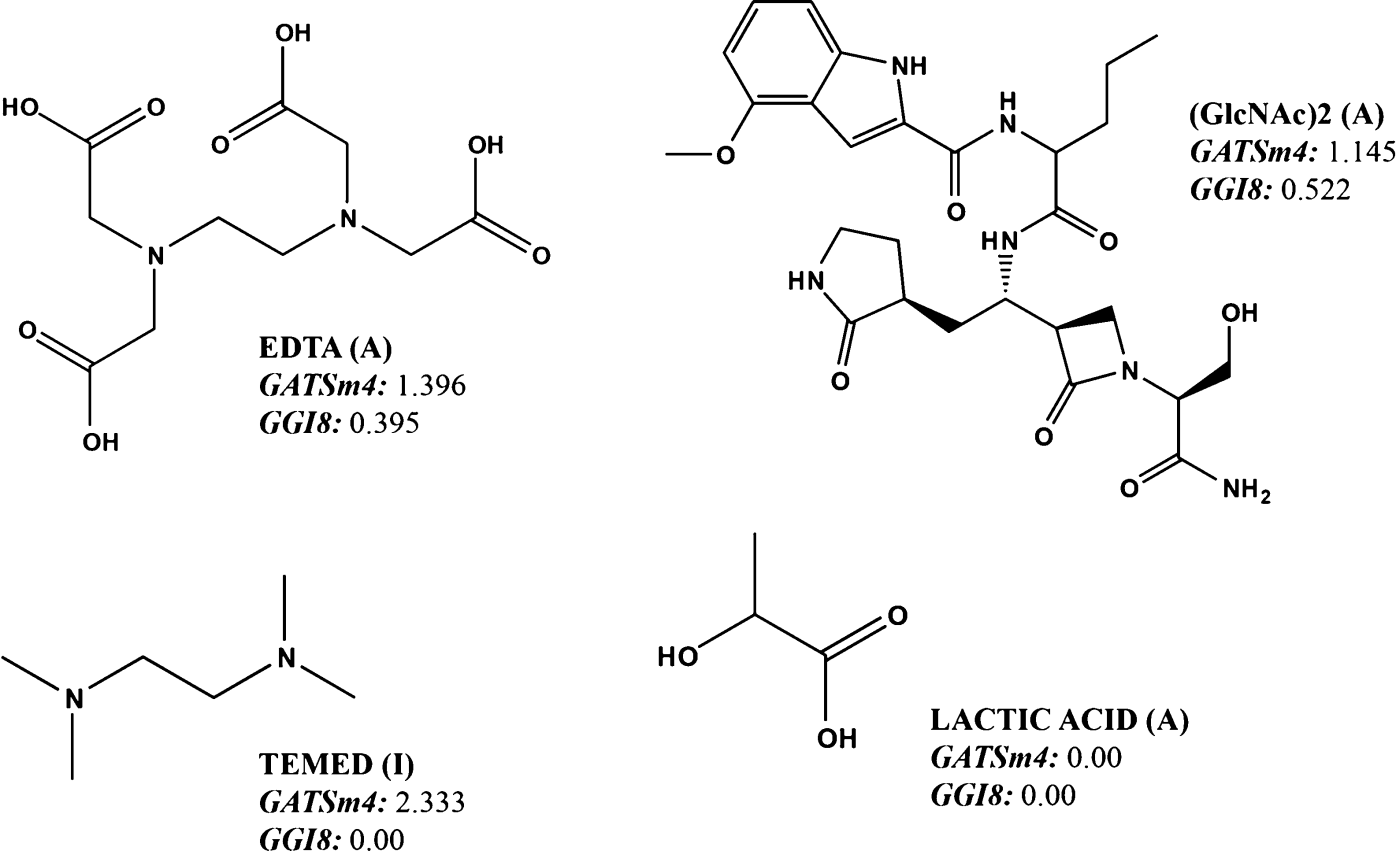
Example of TI values for active (A) and inactive (I) molecules in the DF_1_ training set.

A compound will be classified as active if DF_1_ > 0 and as inactive if DF_1_ < 0. Table S1 shows the DF_1_ values for each training set compound. As can be seen, both the sensitivity and specificity of the DF_1_ function are 100%. In the equation, GATS4m is defined as the Geary autocorrelation—lag4/weighted by atomic mass index and GGI8 is the topological charge index of order 8. GATS4m adopts a negative value in the equation, so the higher the values of this index the lower the activity as a CDA inhibitor. To exemplify it, we can consider the average value of this index for the active group, which is 0.6, while for the inactive group, it is 1.6. GATS4m also considers the presence of different atoms at a distance of 4; hence, compounds such as lactic acid adopt a value of 0 for this descriptor (because none of their atoms are at a distance of 4 between them), while compounds such as TEMED have higher values, because of their greater number of atoms at distance 4. The other descriptor, GGI8 or topological charge index of order 8, exhibits a positive value in the equation so that *a priori*, higher values mean high inhibitory activity against CDA. However, when comparing the index values for the active and inactive training sets, it seems to be not that determinant, because only two compounds show values different from zero in the active group. However, it is not surprising, since this index takes into consideration the total charge transfers between pairs of atoms at distance 8. As it can be seen in Figure 2, only EDTA and (GlcNAc)2 have the presence of atoms at distance 8, so we can affirm that the net charge transfers between atoms at a distance 8 affect the inhibitory activity *versus* CDA, but the charge distribution inside the molecule is not the only factor.

**Figure 3:**
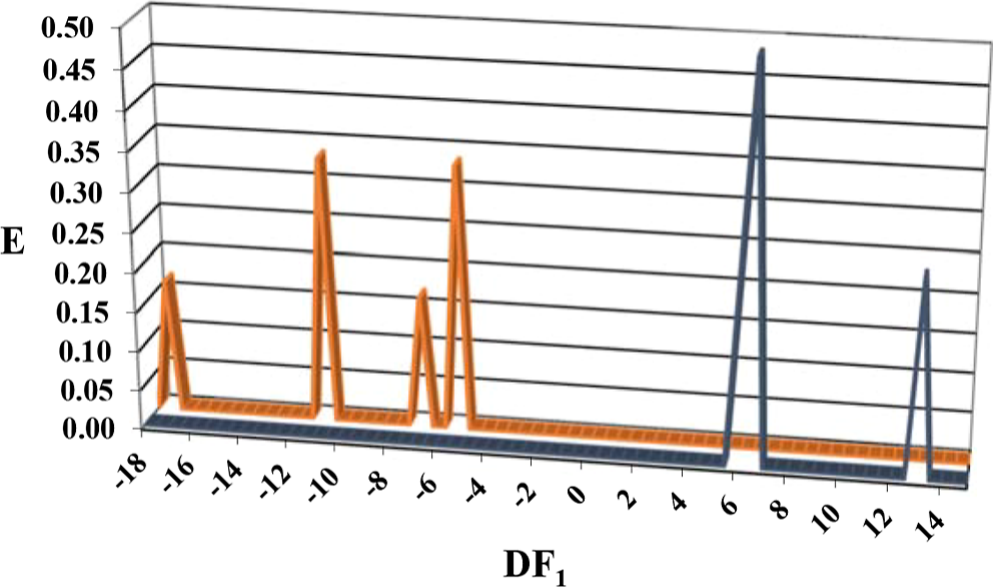
Discriminant function 1 (DF_1_) fungicidal distribution diagram (FDD). Blue peaks represent the CDA inhibitor distribution, while orange peaks the inactive.

As reported in Figure 3, according to the DF_1_, CDA inhibitors (active compounds) had a clear distribution in the range of 0 and +14. Therefore, in this range, compounds are going to be classified as potential CDA inhibitors.

The DF_1_ function was internally validated using a LOO procedure, as the low number of training set compounds (*n* = 10) is unsuitable for performing an external validation of the model. As can be seen in Table S1, results obtained after the internal validation was similar to those of the selected model (see values of DF_1_ and probability of activity). Therefore, the model demonstrates its robustness, and its predictions do not depend on the presence of any single compound in the training set. Once the first discriminant equation (DF_1_) was developed, several virtual libraries of chemicals were screened, such as SPECS, ChEMBL, and eMolecules,^49−51^ searching for novel potential fungicide compounds. Molecules showing values of DF_1_ between 0 and +14 were considered potential CDA inhibitors, while molecules showing values outside this range were considered inactive. In Table S2A, the first set of potential candidates is reported [virtual screening number 1 (VS#**1**) compounds]. Decision-making for the final candidates to be tested *in vitro* was made based on a rate of price/availability and the chemical profile of the substances. Finally, *in vitro* tests were carried out to determine their capability in reducing fungal growth, as an indirect measure of CDA inhibitory activity. According to the experiments, most of the compounds with DF_1_ values between 0 and 14 were experimentally active against *P. xanthii* (see Table S2B), where the inhibitory capacity of the molecules is reported in terms of the percentage of experimental inhibition [Inh(%)(exp)]. The *in vitro* experimental procedures to determine the fungicidal activity are reported in the Materials and Methods section, and the results are further detailed below in the Validation of Fungicide Mode of Action by Molecular Docking section. The experimental biological data were used to develop a MLRA to predict the CDA inhibitory activity of the potential *in vitro* tested candidates. A second predictive equation, based on MLRA, was built up to predict the quantitative CDA inhibitory activity of the assay compounds selected in virtual screening 1 (VS#**1**).

**Figure 4:**
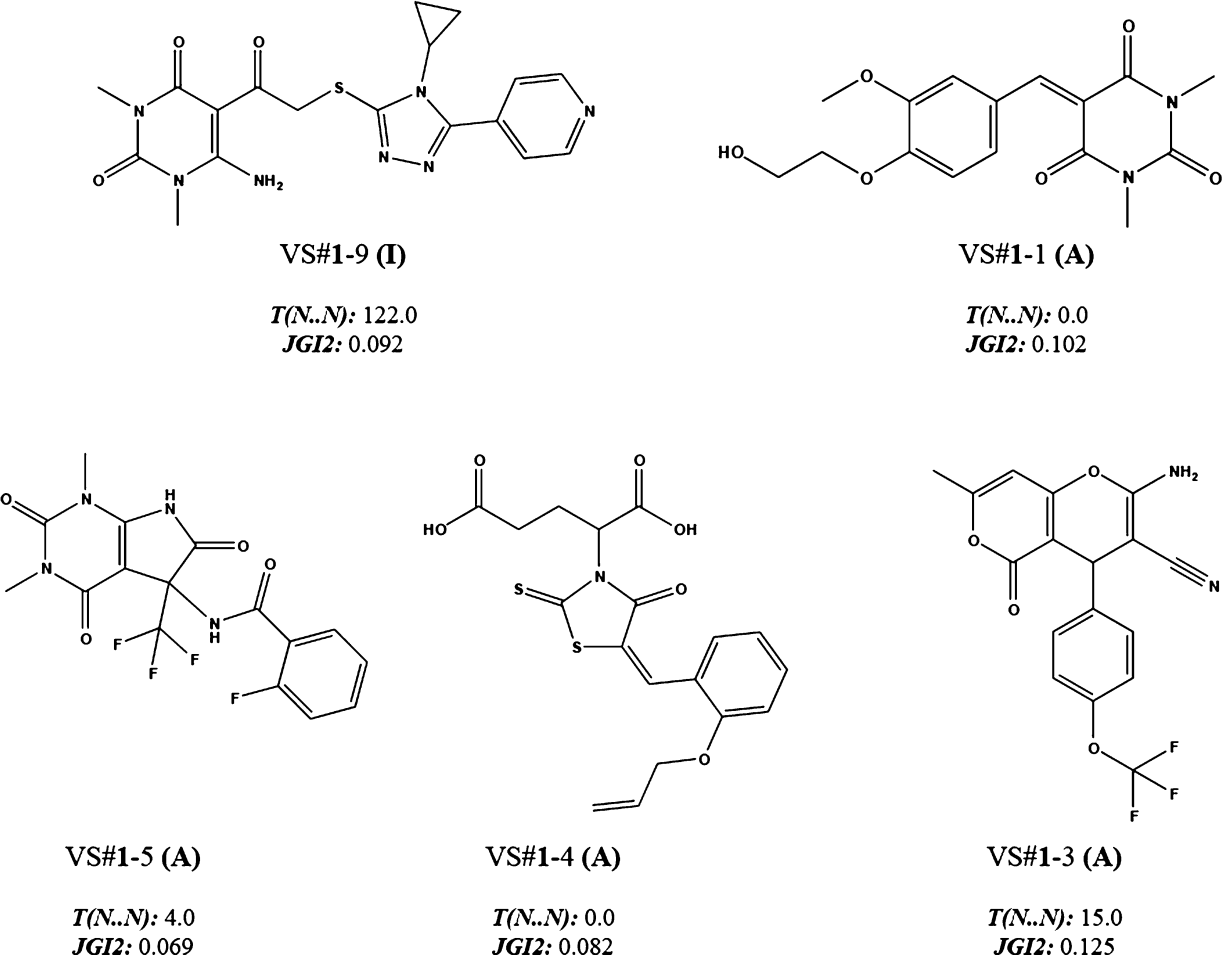
Example of TI values for active (fungicidal activity) and inactive (no fungicidal activity) molecules in the logInh % training set.

The correlation function was
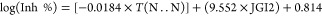
2
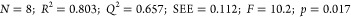
where *N* is the number of VS#1 compounds with experimental fungicidal activity; *R*^2^ is the coefficient of determination; *Q*^2^ is the cross-validation coefficient of determination; SEE is the standard error of estimate; *F* is the Fisher–Snedecor parameter; and *p* is the statistical significance. The descriptors characterizing the regression equation are *T* (N..N), sum of topological distances between N..N, which is the topological distance expressed, and number of edges between two consecutive nitrogen and JGI2 or the mean topological charge index of order 2. The index *T* (N..N) belongs to the topological atom pair descriptors and describes pairs of nitrogen atoms (summing topological distances between all pairs of nitrogen atoms) and bond types connecting them. The two considered atoms need to be not directly connected, and the separation can be the topological distance between them. In Figure 4, it can be seen how the compound VS**#1**-9 adopts value 122, since it has a greater presence of nitrogen atoms at different topological distances, while the molecule VS**#1**-1 adopts value 0 for this index since nitrogen atoms are directly connected, or molecule VS**#1**-4 that does not have any pair of nitrogen atoms in its structure. The negative regression coefficient of this descriptor suggests that a closer topological path between N and N in the structural frame would be better for inhibitory activity. JGI2, the mean topological charge index of order 2, evaluates the global charge transferred between pairs of atoms inside the molecule at two atoms topological distance. This descriptor considers Pauling’s atom electronegativity; therefore, molecules whose atoms have higher Pauling’s electronegativity at a topological distance of 2 should gain higher value for this index. Examples are VS**#1**-3 and VS**#1**-1, which have greater number of N, O, or F atoms at topological distance 2 compared to VS**#1**-5 with a lesser overall presence of atoms with higher Pauling’s electronegativity. Even if a direct relationship between this descriptor and the inhibitory activity against CDA cannot be established, we can get a glimpse that compounds with values above 0.1 (JGI2) are related to fungicidal activity greater than or equal to 50% (see Figure 4, compounds VS**#1**-3 and VS**#1**-1).

**Figure 5:**
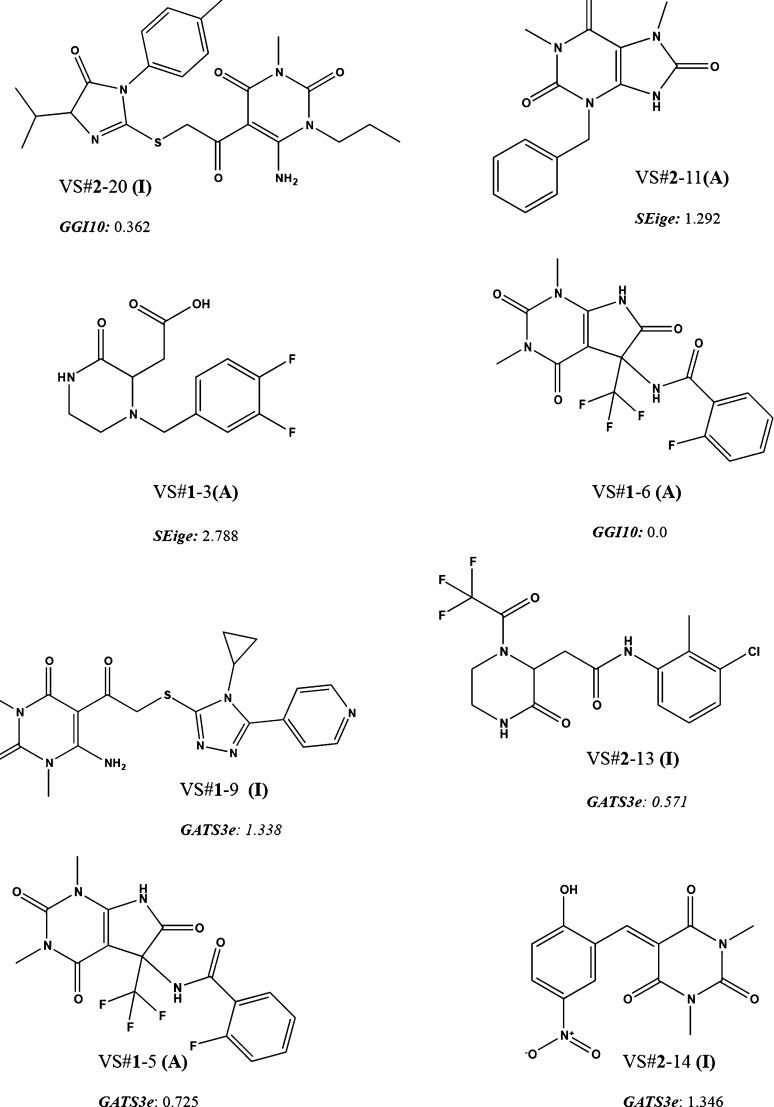
Example of TI values for active and inactive molecules in the logInh % training set.

Again, since the starting data set was small (*n* = 8), internal validation or cross-validation with the LOO procedure to test the model’s quality and reliability was used. The value of prediction coefficient, *Q*^2^ = 0.657, indicates the quality of the prediction function selected.^52^ In Table S3, the value of each descriptor, calculated and observed of log(Inh %) for reported CDA inhibitory activity (indirect measure as fungicidal activity experimentally tested *in vitro*) compounds from VS**#1** are reported. When analyzing results reported in Table S3, a chemo-mathematical pattern for CDA inhibition activity can be seen, as molecules showing a calculated log (Inh %) higher than 1.7 were the ones that showed an experimental fungicidal activity higher than 50%. However, setting such a strict threshold could contribute to the loss of some valuable candidates. Therefore, our cutoff point when applying eq 2 for a virtual screening has been settled in 0.7 log Inh(%) cal, corresponding to at least 5% fungicidal activity. In this way, the greater structural diversity of potential CDA inhibitors can be considered when applying this model. A second virtual screening (VS#**2**) is then carried out using both the first model (qualitative prediction), with the ability to classify CDA inhibitors as active/inactive, and the second topological function (quantitative prediction), with the capability to predict log inhibition. This time, a compound will be selected as a potential CDA inhibitor when showing discriminant values DF_1_ between 0 and 14 and calculated inhibitory activity values log (Inh %) between 0.7 and 2. This chemo-mathematical strategy could lead to potential identification of novel CDA inhibitors based on our experimental results (see Tables S2B and S3), as all compounds showing a certain CDA inhibitory activity (indirect measure as fungicidal activity) follow this pattern. In Table S4A, the list of the compounds chosen after the second virtual screening (VS#**2**) is reported. The values of DF_1_ and log(Inh %)calc, as well as the values of the TIs for each molecule, are reported (Table S4B). As can be seen, the last eight molecules of Table S4B did not show any activity in the experimental settings (0 or equal to 3% of CDA inhibition), whereas half of the selected VS#**2** (10/20) exhibits at least 20% of fungicidal activity. At this point, a valuable in-house experimental data [log Inh (%)] on potential CDA inhibitors selected through the MT strategy were available. Therefore, a small database of compounds with experimental log Inh (%) values was obtained. This information has been parametrized and modeled in the discriminant function number 2 development, where inactive experimentally tested compounds as CDA inhibitors (percentage of fungal growth inhibition equal to or less than 3), and active ones (percentage of fungal growth inhibition equal to or higher than 9) train a model capable of classifying potential CDA inhibitors taking into account experimental *in vitro* test information (VS #1 and #2). The second discriminant equation (DF_2_) was
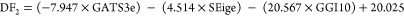
3
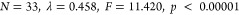
wherein *N* is the number of data compounds, *F* is the Fisher–Snedecor parameter, λ is Wilks’ lambda, and *p* is the statistical significance. The topological descriptors herein are GGI10 or topological charge index of order 10, SEige: eigenvalue sum from electronegativity, and GATS3e: Geary autocorrelation −lag3/weighted by atomic Sanderson electronegativities. GGI10, the topological charge index of order 10 adopts a negative value in the equation, so higher values should be related to low inhibitory activity against CDA. This is not surprising, since the index evaluates the total charge transfer between pairs of atoms at distance 10. In Figure 5, the compounds VS#**2**-20 with the presence of atoms at distance 10 and VS#**1**-6 with minimal presence of atoms at topological distance 10 are reported. The net charge transfers between atoms at distance 10 contribute to a less inhibitory activity *versus* CDA; therefore, a higher charge distribution at distance 10 inside the molecule relates to potential activity. However, this index must not be the only factor affecting the inhibitory activity *versus* CDA, as there is not a direct correlation between this descriptor and the property modeled. Nevertheless, it is interesting to point out that compounds with GGI10 values greater than 0.165 have the highest probability of being inactive as CDA inhibitors. Therefore, there seems to be an optimal charge transfer between atoms that contributes to the biological activity against CDA. Molecules with more compact or non-linear structures exhibit a lower value of this index, so a higher probability of being active may favor interaction with the binding pocket of the enzyme, since the molecules that adopt a zero or low value for this descriptor have a more compact and less elongated design (linear structures). GGI10 tends to increase when the linearity in a molecule increases as it considers atoms at topological distance 10. On the other hand, SEige descriptors are the eigenvalue sum of the electronegativity weighted distance matrix and exhibit a good correlation with the number of hydrogen bonding acceptor atoms (N, O, and F). The higher the presence of these atoms in a molecule, the greater the value for this descriptor, as can be seen in Figure 5—for the compound VS#**1**-3, SEige = 2.788, whereas for VS#**2**-11, SEige = 1.292. Nevertheless, it is not possible to establish a direct relationship between this index and the inhibitory activity *versus* CDA because molecules with smaller eigenvalue sums would not lead to better activity. The last descriptor of the second discriminant equation is GATS3e, Geary autocorrelation −lag3/weighted by atomic Sanderson electronegativities. Indeed, the presence of atoms with higher Sanderson electronegativity such as F or Cl is related to lower values for this index; see molecules in Figure 5 VS#**2**-13 (GATS3e = 0.571) and VS#**1**-5 (GATS3e = 0.725); whereas compounds showing more high values for this index such as VS#**1**-9 (GATS3e = 1.338) and VS#**2**-14 (GATS3e = 1.346) have less presence of halogen at topological distance 3. As GATS3e contributes negatively to the equation, the highest value for this descriptor directly correlates with CDA inhibitory capacity.

Table S5A shows the list of the VS#**1** and VS#**2** selected molecules acting as the training set for the construction of DF_2_ equation, descriptor and discriminant function values, classification by the model, and probability of being classified as a potential CDA inhibitor. According to our last model, a compound will be chosen as a CDA inhibitor if it shows a DF_2_ value between 0 and 6.

**Figure 6:**
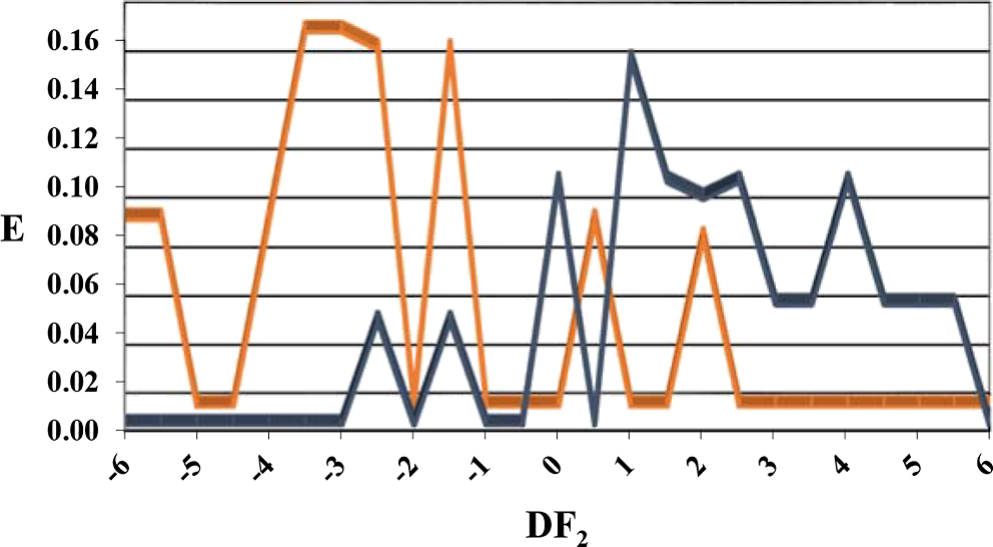
Discriminant function 2 (DF_2_) fungicidal distribution diagram (FDD). Blue peaks represent the distribution of CDA inhibitors, while orange peaks are the ones of non-CDA inhibitors.

In Figure 6, the FDD for DF_2_ is reported. Values over 6 and below −6 will be considered non-classifiable by the model. DF_2_ was validated by following the “leave some out” method; as can be seen in Table S5B, the rate of success in classifying the test set subgroups was higher than 84%. Therefore, the robustness and predictive capability of the model seem to not rely on the presence of a specific group of compounds in the training data set. With all the information gathered, thanks to the three QSAR topological equations, the last VS was carried out (VS#**3**). Taking into account results reported in Table S5A, a potential CDA inhibitor (fungal growth % inhibition equal to or higher than 55) needs to accomplish three chemo-mathematical patterns: DF_1_ value between 0 and 14, a log Inh(%) cal between 0.7 and 2, and finally, a DF_2_ value between 0 and 6. Therefore, these criteria were applied to the final selection of potential CDA inhibitors (VS#**3** compounds) and 10 potential fungicides were selected for further experimental tests. Compounds, along with their structure and the predictive value obtained for each equation (DF_1_, log Inh, and DF_3_), as well as the value of the topological descriptors, are reported in Table S6A,B.

#### 3.1.2 Experimental Strategy and Validation of Compounds Identified by the Computational Approach

##### 3.1.2.1 Determination of Fungicidal Activity by Leaf Disc and Seedling Assays

**Figure 7:**
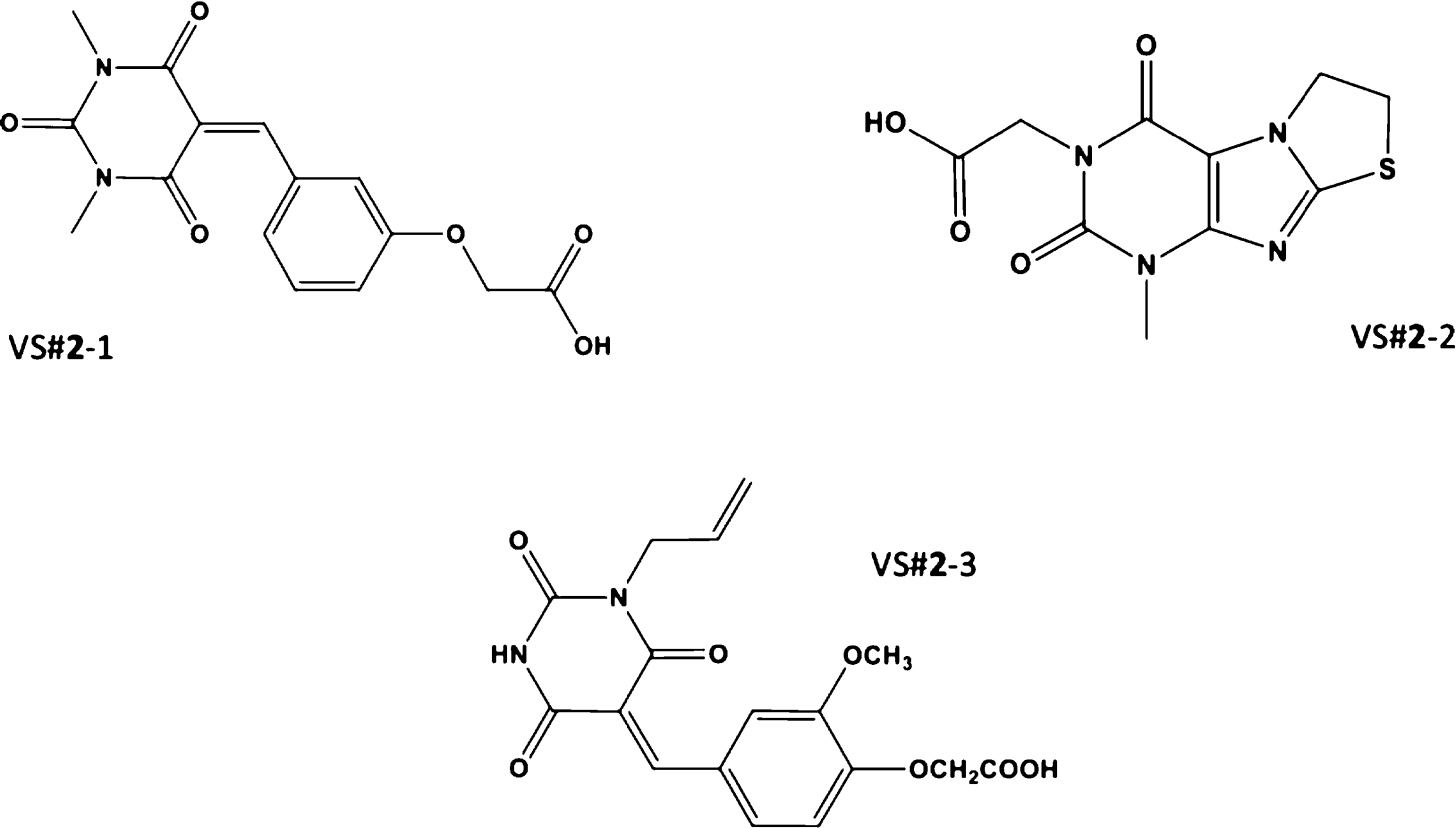
Final selection of potential CDA inhibitors for further experimental validation based on the inhibition of *P. xanthii* development.

Compounds identified by MT screenings were first tested for fungicidal activity on zucchini cotyledon discs against two isolates of *P. xanthii*, isolates 2086 and SF60. In Table S7A, the fungicidal effect of the compounds identified by the MT approach on cucurbit powdery mildew (*P. xanthii*) development in the leaf disc assay is reported. Various compounds showed an efficacy above 50% in reducing disease symptoms compared to the negative control (water). However, the response was different depending on the fungal strain and the concentration used. The leaf disc assay is a good assay for the first screening of the efficacy of fungicidal compounds against powdery mildew.^3^ However, the predicted fungicidal activity of these compounds is not presumably associated with their toxicity but with their ability to activate chitin-triggered immunity, as previously shown for EDTA,^28^ the lead compound used in this study. Since for the efficacy of this response the inoculum distribution can be crucial, we decided to test the compounds by a plant assay using a dispersed inoculum.^44^ Therefore, a seedling assay was subsequently performed using strain 2086 and only one concentration (100 μM). Table S7B shows the fungicidal effect of the compounds identified by the MT approach on the development of the cucurbit powdery mildew *P. xanthii*. In general, the efficacy of the compounds increased compared to the leaf disc assay, and the compounds with the highest fungicidal activity on *P. xanthii* are VS#**2**-1, VS#**2**-2, and VS#**2**-3 (Figure 7).

##### 3.1.2.2 Determination of the Fungicidal Activity of the Three Best Candidates against Three Major Fungal Diseases

**Figure 8:**
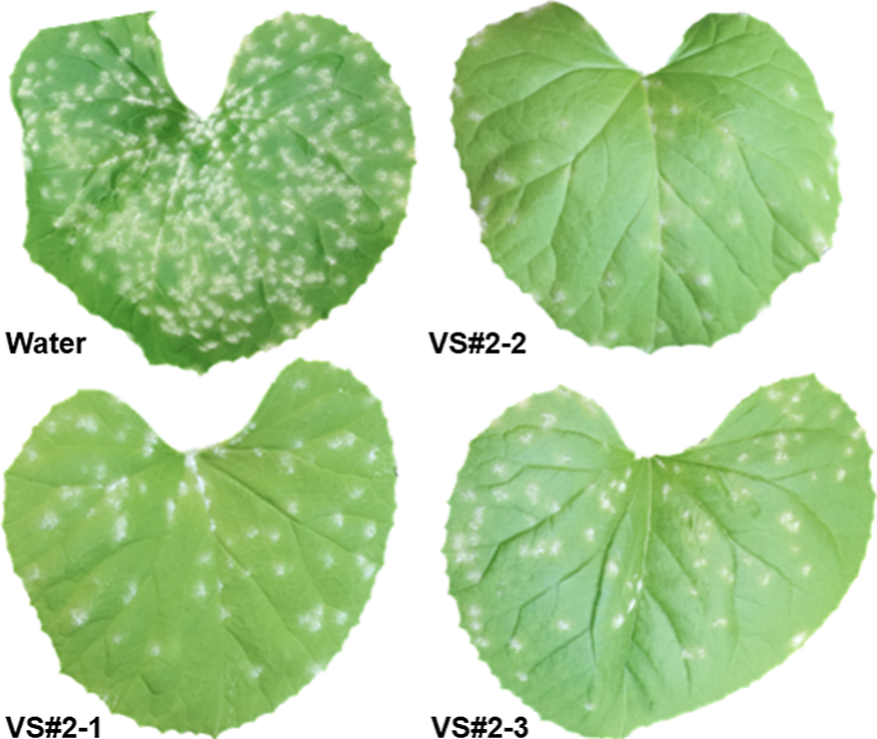
Fungicidal effect of the selected compounds against the cucurbit powdery mildew *P. xanthii*.

The compounds with best potential as fungicides according to the seedling assay were further tested by plant and fruit assays to determine their fungicidal potential against three major fungal diseases, tomato gray mold (*B. cinerea*), citrus green mold (*P. digitatum*), and cucurbit powdery mildew (*P. xanthii*). For post-harvest diseases, assays were conducted using the corresponding fruits.^45,46^ For cucurbit powdery mildew, assays were performed using 6 week old melon plants.^44^ As shown in Table 1, the three compounds showed outstanding disease suppression effects against *P. xanthii* and *B. cinerea*, while against *P. digitatum,* only VS#**2**-3 showed a significant inhibitory effect. As an example, Figure 8 shows the fungicidal effect of the selected compounds against the cucurbit powdery mildew *P. xanthii*. Note how melon leaves show a strong reduction in the number of powdery mildew colonies in leaves treated with the selected compounds.

**Table 1:** Fungicidal Effect of the Selected Compounds in Plant and Fruit Assays against Three Major Plant Fungal Diseases, Cucurbit Powdery Mildew (*P. xanthii*), Tomato Gray Mold (*B. cinerea*), and Citrus Green Mold (*P. digitatum*)

	*P. xanthii* (melon plants)		*B. cinerea* (tomatoes)		*P. digitatum* (oranges)	
compounds^a^	mean^b^	efficacy	mean	efficacy	mean	efficacy
water	39.57 a^c^		89.83 a		100 a	
acetone (1%)	40.77 a	0^d^	87.55 a	2.54	100 a	0
EDTA (20 mM)	3.69 b	90.67	0 c	100	85.22 b	14.78
fluopyram	0 c	100	0 c	100	0 d	100
VS**#2**-2	2.97 b	92.49	0 c	100	99.43 a	0.57
VS**#2**-1	7.51 b	81.02	0 c	100	100 a	0
VS**#2**-3	4.14 b	89.53	0 c	100	58.63 c	41.37

a The compounds and the fungicide fluopyram were applied using solutions at 100 μM.

b The values represent the leaf or fruit surface area covered by fungal growth.

c Different letters are significantly different at *P* = 0.05 according to Fisher’s least significant difference test (LSD).

d The values represent the percentage of the efficacy of the compounds according to Abbott’s formula.

To separate the plant-mediated fungicidal activity from the fungicidal activity *in vitro* (toxicity) of the compounds used in the fruit assays (VS#**2**-1, VS#**2**-2, and VS#**2**-3), the toxicity of such compounds was tested *in vitro* against *B. cinerea* and *P. digitatum* using a microplate assay.^47^ In this assay, fungi were grown in liquid medium in 24-well plates, which were supplemented with the compounds at different concentrations. No inhibitory effect was observed in the range of concentrations tested (Figure S1), suggesting that the fungicidal activity of the compounds is probably related to the activation of plant immunity upon plant detection of non-deacetylated chitin oligomers due to inhibition of fungal CDA, as previously suggested for EDTA.^28^

##### 3.1.2.3 Determination of the Mode of Action as CDA Inhibitors

The compounds with the best fungicide potential were analyzed further to determine their mode of action as CDA inhibitors. Two different experiments were conducted to provide direct (enzymatic assay) and indirect (plant assay) evidence of inhibition of a fungal CDA. First, we attempted to provide evidence for quantitative inhibitory activity by determining *K*_i_ values for the three selected compounds. For this purpose, a CDA activity assay was conducted using the fluorescamine method previously described.^13^ For this assay, two CDA proteins identified in *P. xanthii*, PxCDA1 and PxCDA2,^28^ were expressed *in vitro* in *E. coli* using standard procedures^48^ and exposed to different concentrations of the selected compounds. Unfortunately, the viability of both proteins was very short to construct proper inhibition graphs. However, we did observe some inhibitory effect at 10 and 100 μM. Table 2 shows the results of these experiments. At 100 μM, the compounds induced a strong inhibition of the enzymatic activity of both CDA proteins ranging from 75 to 93%, indicating that those compounds are indeed CDA inhibitors. These compounds were *ca.* 200 times more active than the inhibitory activity of EDTA on the same CDA proteins under similar assay conditions.^54^

**Table 2:** Inhibition of CDA Enzymatic Activity by the Selected Compounds

			inhibition (%)	
compounds	concentration (μM)	reaction time (min)	PxCDA1^a^	PxCDA2
VS#**2**-2	10	45	79.6^b^	83.8
	100	45	79.4	93.0
VS#**2**-1	10	45	64.3	83.7
	100	45	86.9	74.5
VS#**2**-3	10	45	34.3	74.5
	100	45	86.8	74.5

a Two *P. xanthii* CDA enzymes, PxCDA1 and PxCDA2, were expressed *in vitro* in *E. coli* and exposed to the selected compounds.

b The enzymatic activity was determined using the fluorescamine method using colloidal chitin as a substrate. The results were expressed as percentages of inhibition of enzyme activity.

To provide indirect evidence for CDA inhibition, a plant assay on *CERK1*-silenced leaf tissues was carried out. If the inhibition of fungal CDA *in planta* causes the activation of chitin-triggered immunity and the subsequent suppression of fungal growth, the application of CDA inhibitors to leaf tissues in which the *CERK1* gene is silenced should have no effect on fungal growth because of its inability to efficiently activate chitin signaling. The use of *CERK1*-silenced plants have been particularly useful in characterizing fungal effectors with activity in manipulating chitin-triggered immunity, as those plants can no longer recognize immunogenic chitin oligomers.^13,53^

**Figure 9:**
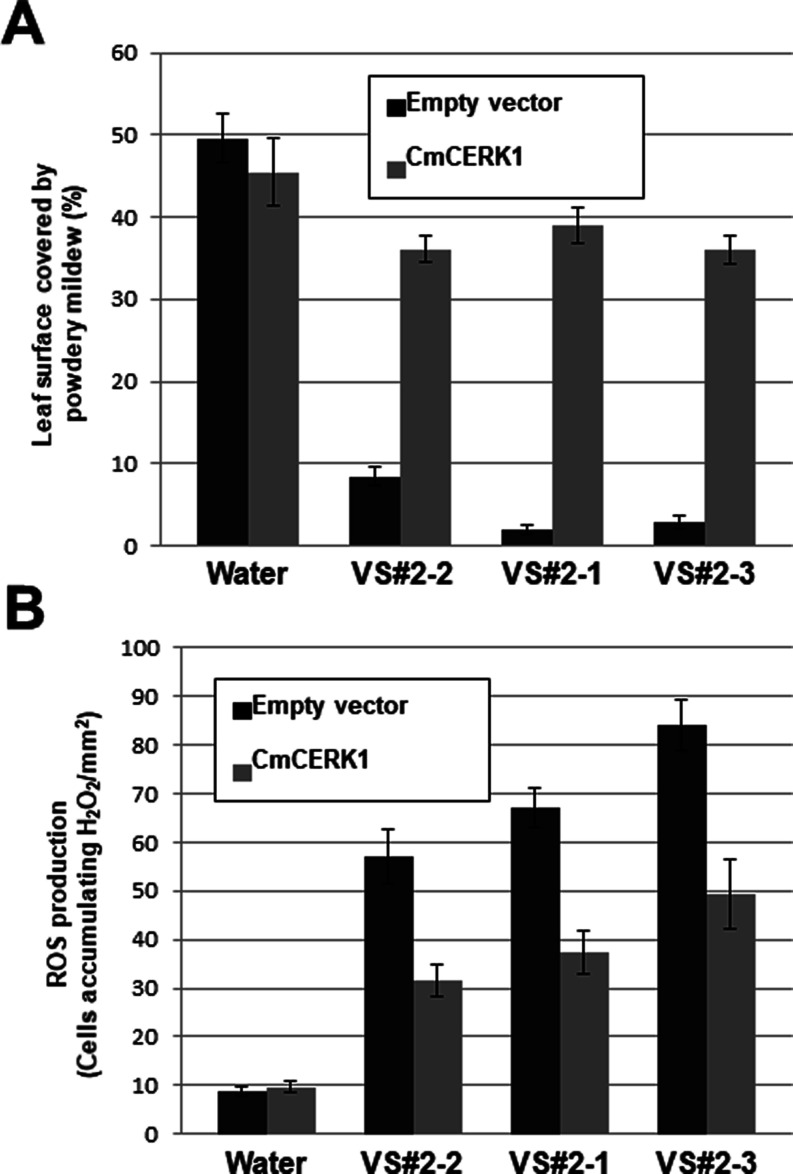
Suppression of fungicidal effects of the selected compounds in *CmCERK1*-silenced melon plants. (A) Suppression of powdery mildew symptoms in *P. xanthii*-infected *CmCERK1*-silenced melon plants treated with the selected compounds. (B) Reduction of oxidative burst (ROS production) after treatment with the selected compounds of *CmCERK1*-silenced melon plants infected with *P. xanthii*.

Figure 9 shows the suppression of the fungicidal effects of the selected compounds when *CERK1*-silenced plants were used. In control plants (empty vector), the compounds induced a strong inhibition of fungal growth (Figure 5A) because of the rapid production of an oxidative burst with the accumulation of reactive oxygen species (ROS) such as hydrogen peroxide (Figure 9B). This plant response was suppressed with the silencing of the chitin receptor *CERK1*. In those plants, fungal growth was restored in presence of the compounds (Figure 9A), and ROS production was reduced (Figure 9B). The results show the relationship between the fungicidal activity of the compounds and the activation of chitin-triggered immunity in the host plant, and they can be explained by the inhibition of CDA activity by the compounds and the subsequent perception of acetylated chitin oligomers by plant receptors, as previously suggested for EDTA.^28^

#### 3.1.3 Validation of Fungicide Mode of Action by Molecular Docking

In order to map the interactions between the best fungicidal compounds and the CDA protein, molecular docking experiments were performed. For these experiments, the entire CDA protein from the causal fungal agent of anthracnose of the common bean *C. lindemunthianum* was used.^42^ As reported in the Materials and Methods section, molecular docking analyses were performed by following standard procedures for a “blind docking” approach, in which the whole CDA protein was analyzed using the SwissDock server. The three best compounds selected from the *in silico* process and the experimental assays (VS#**2**-1, VS#**2**-2, and VS#**2**-3) were docked with CDA protein.

**Figure 10:**
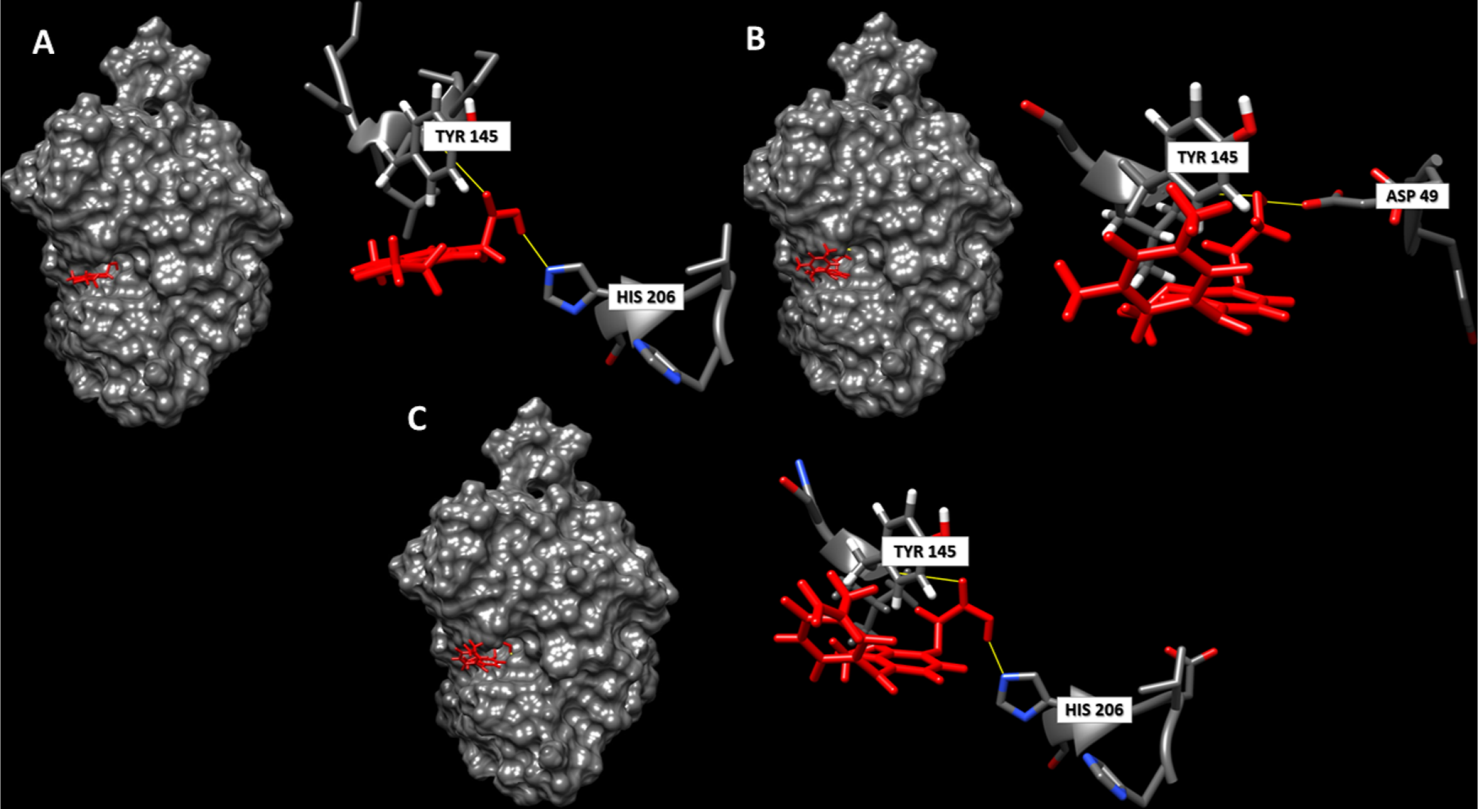
Molecular docking interaction between fungal CDA and potential fungicidal CDA inhibitors: VS#**2**-2 (A), VS#**2**-1(B), and VS#**2**-3 (C).

In Table 3, the results of the docking analysis are reported for the three best candidates and known CDA inhibitors. All the selected potential fungicide (VS#**2**-1, VS#**2**-2, and VS#**2**-3) molecules were able to form at least two hydrogen bonds with specific amino acid residues of the fungal CDA (TYR145, HIS206, and ASP49), thus giving insight into a favorable binding interaction. Furthermore, since TYR145, HIS206, and ASP49 are residues of the potential key catalytic site of the fungal enzyme,^42^ observing the formation of stable H bonds with these residues for VS#**2**-1, VS#**2**-2, and VS#**2**-3 molecules validated its potential mechanism of action on the fungal CDA enzyme. In Figure 10, the interaction of the selected potential fungicides with the fungal CDA of *C. lindemunthianum* is reported.

**Table 3:** Molecular Docking Analysis of a *C. lindemuthianum* CDA Protein

molecular docking analysis of a *C. lindemuthianum* CDA protein^a^					
compound	**residue**	**HB**^b^	**fullfitness(kcal/mol)**	**estimated Δ*G*(kcal/mol)**	**HB distance(Å)**
EDTA	TYR145	2	–699.36	–8.80	1.737
					2.955
lactic acid	TYR145	1	–780.43	–7.44	1.832
propionic acid	TYR145	1	–813.73	–7.05	2.623
	ASP49				1.816
(GlcNAc)_2_	ARG95	1	–675.96	–6.80	2.366
VS#2-2	TYR145	2	–881.24	–7.05	1.801
	HIS206				2.379
VS#2-1	TYR145	2	–823.32	–7.50	2.428
	ASP49				2.371
VS#2-3	TYR145	2	–860.26	–9.08	1.787
	HIS206				2.091

a The protein structure 2IW0 retrieved from the Protein Databank (PDB) was used.

b HB, hydrogen bonds.

**Figure 11:**
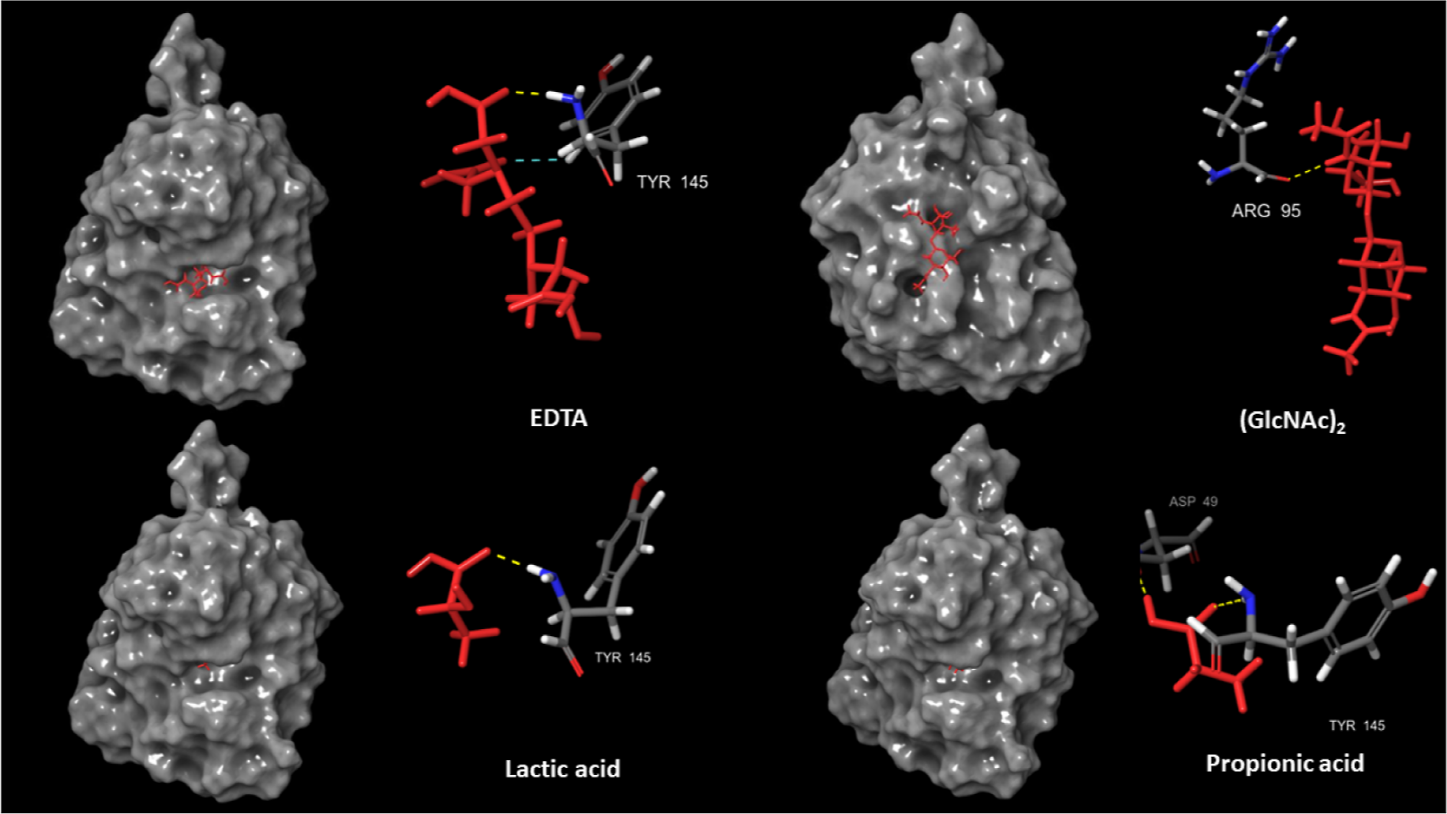
Molecular docking interaction between fungal CDA and known CDA inhibitors: EDTA, (GlcNAc)_2_, lactic acid, and propionic acid.

Finally, if we compare the docking score values obtained for known CDA inhibitors (EDTA, lactic acid, (GlcNAc)_2_, and propionic acid) with the values of the selected potential CDA inhibitors, we found a comparable or even more favorable docking values, as is the case of VS # 2-3 compound. In addition, in Figure 11, it is possible to see how three out of four known CDA inhibitors interact on the same binding site (EDTA, lactic acid, and propionic acid). When analyzing the interactions that strengthen these CDA inhibitors with the fungal CDA enzyme, they interact through hydrogen bonds with at least two of the three AAs reported as key AAs of the binding catalytic pocket (TYR145 and ASP49).^42^ Finally, TYR145 seems to play a key role in the inhibition of CDA, since three of the known CDA inhibitors and the potential CDA inhibitors selected by MT interact with this amino acid.

Taking together, the molecular docking results provided computational evidence in support of the ability of the selected molecules to modulate the CDA enzyme, thus reinforcing their activities as specific CDA inhibitors.

**Figure 12:**
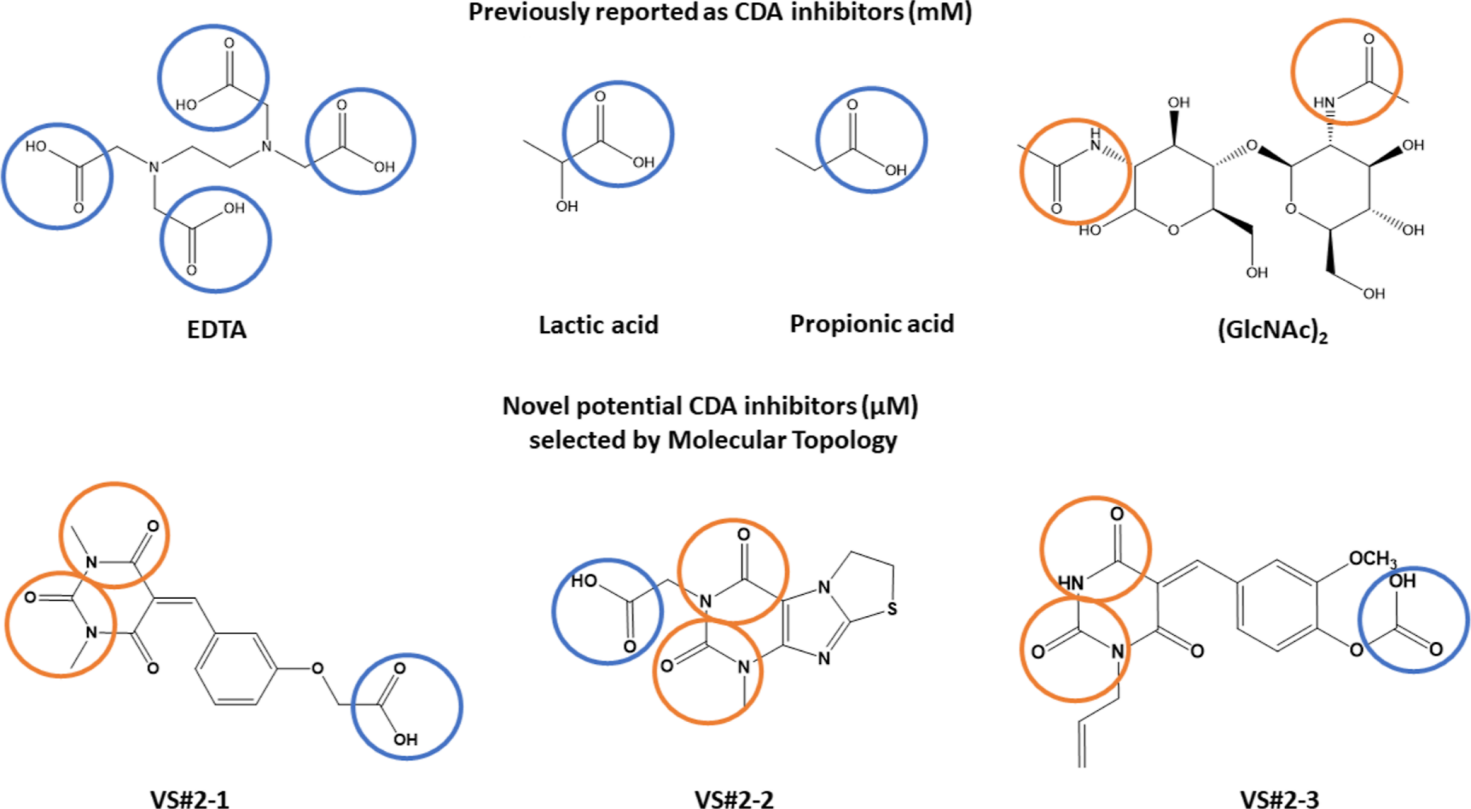
New CDA inhibitors designed through the MT approach.

In conclusion, starting from a very small data of only ten compounds, whose activity on CDA was described in the literature^42^ and further corroborated through our biological assays,^55^ a MT-based computational strategy was developed, which allowed us to identify the chemo-mathematical pattern for their inhibitory activity against CDA. Thus, starting from four molecules with reported inhibitory activity against CDA (EDTA, (GlcNAc)_2_, lactic acid, and propionic acid), most of the carboxylic acids have CDA inhibitory activity at a concentration of millimoles; new molecular scaffolds had been discovered, capable of exerting inhibitory activity of CDA at a way lower concentration range (μM). As it can be appreciated in Figure 12, for our novel CDA inhibitors VS#**2**-1, VS#**2**-2, and VS#**2**-3, structural similarities to both carboxylic acids and (GlcNAc)_2_ are present, since they had acid and amide groups in their structures. This pattern can be appreciated by means of molecular docking, as known CDA inhibitors (Figure 11) establish interactions with CDA amino acids by interacting with acid (EDTA and lactic and propionic acids) and amide (GlcNAc)_2_ functional groups.

Therefore, topological models have been able to identify the key chemical structural features needed to express and identify CDA inhibitory activity and design a novel generation of CDA inhibitors. This is explained as that the MT approach allows a totally different kind of similarity search between active molecules, based on a mathematical paradigm that translates chemical structures into topological information, by means of topological descriptors and allows the identification of candidates which may be completely different structurally but share the same exact topological pattern. For that reason, it may be possible to find certain similarities between the chemical groups identified by MT and their importance in the interaction between the ligand and the target, but it does not have to be the case.

These compounds are promising because they seem to be capable of stimulating the immune system of the plant by triggering a defensive response against fungal species such as powdery mildew fungi, which are becoming particularly resistant to common fungicides.

Future SAR studies will be performed to enhance the potency of the selected compounds as CDA inhibitors.

## Supporting Information Available

Descriptor values, probability of being classified as active by the model, DF_1_ value for training set compounds, and probability of being classified as active by the internal validated model (LOO); first selection of potential CDA inhibitors (virtual screening number 1, VS#**1**) applying DF_1_ model and first virtual screening results (VS#**1**): selection of potential CDA inhibitors according to DF_1_; calculated log Inh(%) activity for all compounds selected in the first virtual screening (VS#1) and experimental results; second selection of potential CDA inhibitors (virtual screening number 2, VS#**2**) applying eqs 1 and 2 and a list of compounds chosen after the second virtual screening (VS#**2**); value of DF_1_ and log(Inh %)calc, as well as the values of TIs; descriptor values, DF_2_ value for training set compounds, probability of being classified as a potential CDA inhibitor, classification as active or inactive by the model, and quantitative and qualitative experimental values related to fungicidal activity; LSO internal validation procedure for DF_2_ ; third selection of potential CDA inhibitors (virtual screening number 3, VS#**3**) and virtual screening number 3, applying DF_1_, log Inh, and DF_2_; fungicidal effect of compounds identified by the MT approach on cucurbit powdery mildew development (*P. xanthii*) in the leaf disc assay; fungicidal effect of compounds identified by the MT approach on the cucurbit powdery mildew *P. xanthii*; and *in vitro* antifungal activity of compounds identified by MT (PDF).

## References

[ref1] Vielba-Fernández A.; Polonio Á.; Ruiz-Jiménez L.; de Vicente A.; Pérez-García A.; Fernández-Ortuño D. Fungicide resistance in powdery mildew fungi. Microorganisms 2020, 8, 1431 10.3390/microorganisms8091431. 32957583 PMC7564317

[ref2] Glawe D. A. The powdery mildews: a review of the world’s most familiar (yet poorly known) plant pathogens. Annu. Rev. Phytopathol. 2008, 46, 27–51. 10.1146/annurev.phyto.46.081407.104740. 18680422

[ref3] Fernández-Ortuño D.; Pérez-García A.; López-Ruiz F.; Romero D.; de Vicente A.; Torés J. A. Occurrence and distribution of resistance to QoI fungicides in populations of Podosphaera fusca in south central Spain. Eur. J. Plant Pathol. 2006, 115, 215–222. 10.1007/s10658-006-9014-7.

[ref4] López-Ruiz F. J.; Pérez-García A.; Fernández-Ortuño D.; Romero D.; García E.; de Vicente A.; Brown J. K.; Torés J. A. Sensitivities to DMI fungicides in populations of Podosphaera fusca in south central Spain. Pest Manag. Sci. 2010, 66, 801–808. 10.1002/ps.1948. 20533378

[ref5] Bellón-Gómez D.; Vela-Corcía D.; Pérez-García A.; Torés J. A. Sensitivity of Podosphaera xanthii populations to anti-powdery-mildew fungicides in Spain. Pest Manag. Sci. 2015, 71, 1407–1413. 10.1002/ps.3943. 25418926

[ref6] Vielba-Fernández A.; Bellón-Gómez D.; Torés J. A.; de Vicente A.; Pérez-García A.; Fernández-Ortuño D. Heteroplasmy for the cytochrome b gene in Podosphaera xanthii and its role in resistance to QoI fungicides in Spain. Plant Dis. 2018, 102, 1599–1605. 10.1094/PDIS-12-17-1987-RE. 30673427

[ref7] Vielba-Fernández A.; de Vicente A.; Pérez-García A.; Fernández-Ortuño D. Monitoring methyl benzimidazole carbamate-resistant isolates of the cucurbit powdery mildew pathogen, Podosphaera xanthii, using loop-mediated isothermal amplification. Plant Dis. 2019, 103, 1515–1524. 10.1094/PDIS-12-18-2256-RE. 31059385

[ref8] Gow N. A.; Latge J.; Munro C. A. The fungal cell wall: structure, biosynthesis, and function. Microbiol. Spectr. 2017, 5, 5–301. 10.1128/microbiolspec.FUNK-0035-2016. PMC11687499 28513415

[ref9] Wan J.; Zhang X.; Stacey G. Chitin signaling and plant disease resistance. Plant Signal. Behav. 2008, 3, 831–833. 10.4161/psb.3.10.5916. 19704513 PMC2634388

[ref10] Miya A.; Albert P.; Shinya T.; Desaki Y.; Ichimura K.; Shirasu K.; Narusaka Y.; Kawakami N.; Kaku H.; Shibuya N. CERK1, a LysM receptor kinase, is essential for chitin elicitor signaling in Arabidopsis. Proc. Natl. Acad. Sci. U. S. A. 2007, 104, 19613–19618. 10.1073/pnas.0705147104. 18042724 PMC2148337

[ref11] Kaku H.; Nishizawa Y.; Ishii-Minami N.; Akimoto-Tomiyama C.; Dohmae N.; Takio K.; Minami E.; Shibuya N. Plant cells recognize chitin fragments for defense signaling through a plasma membrane receptor. Proc. Natl. Acad. Sci. U. S. A. 2006, 103, 11086–11091. 10.1073/pnas.0508882103. 16829581 PMC1636686

[ref12] de Jonge R.; Peter van Esse H.; Kombrink A.; Shinya T.; Desaki Y.; Bours R.; van der Krol S.; Shibuya N.; Joosten M. H.; Thomma B. P. Conserved fungal LysM effector Ecp6 prevents chitin-triggered immunity in plants. Science 2010, 329, 953–955. 10.1126/science.1190859. 20724636

[ref13] Mart Nez-Cruz J.; Romero D.; Hierrezuelo J.; Thon M.; de Vicente A.; P Rez-Garc A A. Effectors with chitinase activity (EWCAs), a family of conserved, secreted fungal chitinases that suppress chitin-triggered immunity. Plant Cell 2021, 33, 1319–1340. 10.1093/plcell/koab011. 33793825

[ref14] Gao F.; Zhang B.; Zhao J.; Huang J.; Jia P.; Wang S.; Zhang J.; Zhou J.; Guo H. Deacetylation of chitin oligomers increases virulence in soil-borne fungal pathogens. Nat. Plants 2019, 5, 1167–1176. 10.1038/s41477-019-0527-4. 31636399

[ref15] Zanni R.; Galvez-Llompart M.; García-Domenech R.; Galvez J. Latest advances in molecular topology applications for drug discovery. Expet Opin. Drug Discov. 2015, 10, 945–957. 10.1517/17460441.2015.1062751. 26134383

[ref16] Zanni R.; Galvez-Llompart M.; Garcia-Domenech R.; Galvez J. What place does molecular topology have in today’s drug discovery?. Expet Opin. Drug Discov. 2020, 15, 1133–1144. 10.1080/17460441.2020.1770223. 32496823

[ref17] Galvez J.; Garcia-Domenech R. On the contribution of molecular topology to drug design and discovery. Curr. Comput.-Aided Drug Des. 2010, 6, 252–268. 10.2174/1573409911006040252. 20883200

[ref18] García-Domenech R.; Gálvez J.; de Julián-Ortiz J. V.; Pogliani L. Some new trends in chemical graph theory. Chem. Rev. 2008, 108, 1127–1169. 10.1021/cr0780006. 18302420

[ref19] Carbó-Dorca R. Shadows’ hypercube, vector spaces, and non-linear optimization of QSPR procedures. J. Math. Chem. 2022, 60, 283–310. 10.13140/RG.2.2.16118.93764.

[ref20] Basak S. C.; Balaban A. T.; Grunwald G. D.; Gute B. D. Topological indices: their nature and mutual relatedness. J. Chem. Inf. Comput. Sci. 2000, 40, 891–898. 10.1021/ci990114y. 10955515

[ref21] Galvez J.; Garcia R.; Salabert M. T.; Soler R. Charge indexes. New topological descriptors. J. Chem. Inf. Comput. Sci. 1994, 34, 520–525. 10.1021/ci00019a008.

[ref22] Gálvez-Llompart M.; Gálvez J.; García-Domenech R.; Kier L. B. Predicting dyspnea inducers by molecular topology. J. Chem. 2013, 2013, 1–11. 10.1155/2013/798508.

[ref23] Zanni R.; Galvez-Llompart M.; Morell C.; Rodríguez-Henche N.; Díaz-Laviada I.; Recio-Iglesias M. C.; Garcia-Domenech R.; Galvez J. Novel cancer chemotherapy hits by molecular topology: Dual Akt and Beta-catenin inhibitors. PLoS One 2015, 10, e0124244 10.1371/journal.pone.0124244. 25910265 PMC4409212

[ref24] Galvez-Llompart M.; Ocello R.; Rullo L.; Stamatakos S.; Alessandrini I.; Zanni R.; Tuñón I.; Cavalli A.; Candeletti S.; Masetti M.; Romualdi P.; Recanatini M. Targeting the JAK/STAT Pathway: A Combined Ligand-and Target-Based Approach. J. Chem. Inf. Model. 2021, 61, 3091–3108. 10.1021/acs.jcim.0c01468. 33998810 PMC8491162

[ref25] Galvez J.; Zanni R.; Galvez-Llompart M.; Benlloch J. M. Macrolides may prevent severe acute respiratory syndrome coronavirus 2 entry into cells: a quantitative structure activity relationship study and Experimental validation. J. Chem. Inf. Model. 2021, 61, 2016–2025. 10.1021/acs.jcim.0c01394. 33734704

[ref26] Galvez-Llompart M.; Zanni R.; Galvez J.; Garcia-Domenech R. Molecular Topology QSAR Strategy for Crop Protection: New Natural Fungicides with Chitin Inhibitory Activity. ACS Omega 2020, 5, 16358–16365. 10.1021/acsomega.0c00177. 32685798 PMC7364431

[ref27] Zanni R.; Galvez-Llompart M.; Garcia-Pereira I.; Galvez J.; Garcia-Domenech R. Molecular topology and QSAR multi-target analysis to boost the in silico research for fungicides in agricultural chemistry. Mol. Divers. 2019, 23, 371–379. 10.1007/s11030-018-9879-3. 30284694

[ref28] Martínez-Cruz J. M.; Polonio Á.; Zanni R.; Romero D.; Gálvez J.; Fernández-Ortuño D.; Pérez-García A. Chitin Deacetylase, a Novel Target for the Design of Agricultural Fungicides. J. Fungi 2021, 7, 1009 10.3390/jof7121009. PMC8706340 34946992

[ref29] Pérez-García A.; Martínez-Cruz J.; Zanni R.; Romero-Hinojosa D.; Fernández-Ortuño D.; Galvez-Llompart M.; Garcia-Domenech R.; Galvez J. Chitin deacetylase inhibitors and their use as agricultural fungicides, arthropocides and nematicides. WO 2020,234,497, Spain, 2019.

[ref30] ChemDraw Cousins K. R. ChemDraw Ultra 12.0. CambridgeSoft, 100 CambridgePark Drive: Cambridge, MA, 02140, 2012. CA. Available online:. www.cambridgesoft.com.

[ref31] Roy K. Topological descriptors in drug design and modeling studies. Mol. Divers. 2004, 8, 321–323. 10.1023/b:modi.0000047519.35591.b7. 15612635

[ref32] Mauri A.; Consonni V.; Pavan M.; Todeschini R. Dragon software: An easy approach to molecular descriptor calculations. MATCH Communications in Mathematical and in Computer Chemistry; 2006; Vol. 56, pp 237–248.

[ref33] Solberg H. E. Discriminant analysis. CRC Crit. Rev. Clin. Lab. Sci. 1978, 9, 209–242. 10.3109/10408367809150920. 401370

[ref34] StatSoft, Inc.. STATISTICA (Data Analysis Software System); version 6, StatSoft, Inc.: Tulsa, OK, USA, 2001; Vol. 150, pp 91–94.

[ref35] Gálvez J.; García-Domenech R.; de Gregorio Alapont C.; de Julián-Ortiz J. V.; Popa L. Pharmacological distribution diagrams: a tool for de novo drug design. J. Mol. Graph. 1996, 14, 272–276. 10.1016/s0263-7855(96)00081-1. 9097233

[ref36] Furnival G. M.; Wilson R. W. Regressions by leaps and bounds. Technometrics 2000, 42, 69–79. 10.1080/00401706.2000.10485982.

[ref37] Sanchez-Pinto L. N.; Venable L. R.; Fahrenbach J.; Churpek M. M. Comparison of variable selection methods for clinical predictive modeling. Int. J. Med. Inf. 2018, 116, 10–17. 10.1016/j.ijmedinf.2018.05.006. PMC6003624 29887230

[ref38] Hawkins D. M.; Basak S. C.; Mills D. Assessing model fit by cross-validation. J. Chem. Inf. Comput. Sci. 2003, 43, 579–586. 10.1021/ci025626i. 12653524

[ref39] Baumann K. Cross-validation as the objective function for variable-selection techniques. Trac. Trends Anal. Chem. 2003, 22, 395–406. 10.1016/s0165-9936(03)00607-1.

[ref40] Grosdidier A.; Zoete V.; Michielin O. SwissDock, a protein-small molecule docking web service based on EADock DSS. Nucleic Acids Res. 2011, 39, W270–W277. 10.1093/nar/gkr366. 21624888 PMC3125772

[ref41] Pettersen E. F.; Goddard T. D.; Huang C. C.; Couch G. S.; Greenblatt D. M.; Meng E. C.; Ferrin T. E. UCSF Chimera?A visualization system for exploratory research and analysis. J. Comput. Chem. 2004, 25, 1605–1612. 10.1002/jcc.20084. 15264254

[ref42] Blair D. E.; Hekmat O.; Schüttelkopf A. W.; Shrestha B.; Tokuyasu K.; Withers S. G.; van Aalten D. M. Structure and mechanism of chitin deacetylase from the fungal pathogen Colletotrichum lindemuthianum. Biochemistry 2006, 45, 9416–9426. 10.1021/bi0606694. 16878976

[ref43] Jukič M.; Konc J.; Gobec S.; Janežič D. Identification of conserved water sites in protein structures for drug design. J. Chem. Inf. Model. 2017, 57, 3094–3103. 10.1021/acs.jcim.7b00443. 29155577

[ref44] Romero D.; Rivera M. E.; Cazorla F. M.; De Vicente A.; Pérez-garcía A. Effect of mycoparasitic fungi on the development of Sphaerotheca fusca in melon leaves. Mycol. Res. 2003, 107, 64–71. 10.1017/s0953756202006974. 12735245

[ref45] Fernández-Ortuño D.; Chen F.; Schnabel G. Resistance to cyprodinil and lack of fludioxonil resistance in Botrytis cinerea isolates from strawberry in North and South Carolina. Plant Dis. 2013, 97, 81–85. 10.1094/PDIS-06-12-0539-RE. 30722260

[ref46] Holmes G. J.; Eckert J. W. Sensitivity of Penicillium digitatum and P. italicum to postharvest citrus fungicides in California. Phytopathology 1999, 89, 716–721. 10.1094/phyto.1999.89.9.716. 18944698

[ref47] Broekaert W. F.; Terras F. R.; Cammue B. P.; Vanderleyden J. An automated quantitative assay for fungal growth inhibition. FEMS Microbiol. Lett. 1990, 69, 55–59. 10.1111/j.1574-6968.1990.tb04174.x.

[ref48] Martínez-Cruz J.; Romero D.; de la Torre F. N.; Fernández-Ortuño D.; Torés J. A.; de Vicente A.; Pérez-García A. The functional characterization of Podosphaera xanthii candidate effector genes reveals novel target functions for fungal pathogenicity. Mol. Plant-Microbe Interact. 2018, 31, 914–931. 10.1094/MPMI-12-17-0318-R. 29513627

[ref49] Database SPECS SPECS, NV Delft, The Netherlands. Available online. https://www.specs.net/ (May 2021).

[ref50] Gaulton A.; Hersey A.; Nowotka M.; Bento A. P.; Chambers J.; Mendez D.; Mutowo P.; Atkinson F.; Bellis L. J.; Cibrián-Uhalte E.; Davies M.; Dedman N.; Karlsson A.; Magariños M. P.; Overington J. P.; Papadatos G.; Smit I.; Leach A. R. The ChEMBL database in 2017. Nucleic Acids Res. 2017, 45, D945–D954. 10.1093/nar/gkw1074. 27899562 PMC5210557

[ref51] Gupta P. P.; Bastikar V. A.; Chhajed S. S. Chemical Structure Databases in Drug Discovery. In Computer Applications in Drug Discovery and Development; IGI Global, 2019; pp 47–61.

[ref52] Gramatica P. On the development and validation of QSAR models. In Computational toxicology; Anonymous; Springer, 2013; pp 499–526. 10.1007/978-1-62703-059-5_21 23086855

[ref53] Polonio Á.; Fernández-Ortuño D.; Vicente A.; Pérez-García A. A haustorial-expressed lytic polysaccharide monooxygenase from the cucurbit powdery mildew pathogen Podosphaera xanthii contributes to the suppression of chitin-triggered immunity. Mol. Plant Pathol. 2021, 22, 580–601. 10.1111/mpp.13045. 33742545 PMC8035642

[ref54] Martínez-Cruz J. Análisis morfológico y funcional de la interacción Podosphaera xanthii-cucurbitáceas. Ph.D. Thesis, University of Malaga: Malaga, Spain, 2016.

[ref55] Kafetzopoulos D.; Martinou A.; Bouriotis V. Bioconversion of chitin to chitosan: purification and characterization of chitin deacetylase from Mucor rouxii. Proc. Natl. Acad. Sci. U. S. A. 1993, 90, 2564–2568. 10.1073/pnas.90.7.2564. 8464862 PMC46135

