# Supplementary material for: Rational Design of Chitin Deacetylase Inhibitors for Sustainable Agricultural Use Based on Molecular Topology
Source: J Agric Food Chem. 2022 Oct 4;70(41):13118–31. doi: 10.1021/acs.jafc.2c02377 (PMC10389753; doi:10.1021/acs.jafc.2c02377)
Supplement: Supplementary file 1 — jf2c02377_si_001.pdf [file jf2c02377_si_001.pdf]

Supplementary Information

Rational design of chitin deacetylase inhibitors for sustainable agricultural use based on Molecular Topology

Riccardo Zanni[1], Jesús Martínez-Cruz[2,3], María Gálvez-Llompart[1], Dolores Fernández-Ortuño[2,3], Diego Romero[2,3], Ramón García-Domènech[1], Alejandro Pérez-García[2,3], Jorge Gálvez[1]

[1] Molecular Topology and Drug Design Unit, Department of Physical Chemistry, University of Valencia, 46010 Valencia, Spain

[2] Departamento de Microbiología, Facultad de Ciencias, Universidad de Málaga, 29071 Málaga, Spain

[3] Instituto de Hortofruticultura Subtropical y Mediterránea "La Mayora", Universidad de Málaga, Consejo Superior de Investigaciones Científicas (IHSM-UMA-CSIC), 29071 Málaga, Spain

Corresponding Author

Jorge Galvez - *Molecular Topology and Drug Design Unit, Department of Physical Chemistry, University of Valencia, 46010 Valencia, Spain;* orcid.org/0000-0003-0928-8437 ; Phone: 34-6-3544891; Email: jorge.galvez@uv.es

Table S1. Descriptors values, probability of being classified as active by the model, DF_1 value for training set compounds and probability of being classified as active by the internal validated model (LOO).

**Compound**	**GATS4m**	**GGI8**	**P.A.**[b]	**DF_1**[c]	**P.A. (LOO)**[b]
			**Active group**[a]		
EDTA	1.396	0.395	0.9984	**6.42**	0.9843
(GlcNAc)2	1.145	0.522	1.0000	**13.46**	1.0000
Lactic acid	0	0	0.9990	**6.92**	0.9873
Propionic acid	0	0	0.9990	**6.92**	0.9873
			**Inactive group**[a]		
2-Mercaptopropanol	1.707	0	0.0000	**-10.66**	0.0001
8-Quinolinol	1.726	0	0.0000	**-10.85**	0.0001
Citric acid	1.311	0	0.0014	**-6.58**	0.0028
GlcNAc*	1.172	0	0.0058	**-5.15**	0.0085
Hexanoic acid	1.167	0	0.0061	**-5.10**	0.0089
TEMED	2.333	0	0.0000	**-17.10**	0.0000

[a] Experimental classification of the CDA inhibitory activity of the compounds used as starting material to perform the first LDA
[b] probability of being classified each compound as active
[c] Value of the discriminant function, DF1, obtained for each compound ($DF_1 = (-10.295 \times GATS4m) + (35.124 \times GGI8) + 6.917$); where GATS4m, Geary autocorrelation – lag4 /weighted by atomic masses; GGI8, topological charge index of order 8.
* N-Acetilglucosamina

Table S2A. First selection of potential CDA inhibitors (virtual screening number 1, VS#**1**) applying DF_1 model.

Name	Comp. Nº	Srtucture and SMILES code
5-[4-(2-hydroxyethoxy)-3-methoxybenzylidene]-1,3-dimethyl-2,4,6(1H,3H,5H)-pyrimidinetrione	VS#**1**-1	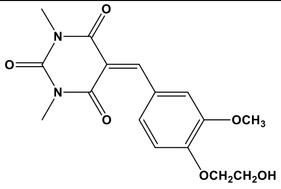 **O=C(N(C)C/1=O)N(C)C(C1=C\C2=CC(OC)=C(C=C2)OCCO)=O**
[4-(1-Allyl-2,4,6-trioxo-tetrahydro-pyrimidin-5-ylidenemethyl)-2-methoxy-phenoxy]-acetic acid	VS#**1**-2	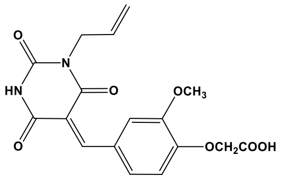 **O=C(NC/1=O)N(CC=C)C(C1=C/C2=CC=C(C(OC)=C2)OCC(O)=O)=O**
N-[1,3-dimethyl-2,4,6-trioxo-5-(trifluoromethyl)-2,3,4,5,6,7-hexahydro-1H-pyrrolo[2,3-d]pyrimidin-5-yl]-2-fluorobenzamide	VS#**1**-3	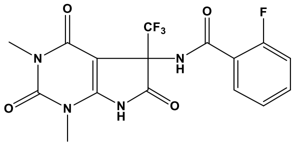 **O=C1N(C)C(C(C2(NC(C3=CC=CC=C3F)=O)C(F)(F)F)=C(NC2=O)N1C)=O**
2-[5-(2-Allyloxy-benzylidene)-4-oxo-2-thioxo-thiazolidin-3-yl]-pentanedioic acid	VS#**1**-4	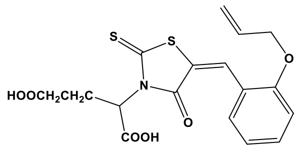 **S=C(S/1)N(C(CCC(O)=O)C(O)=O)C(C1=C/C2=CC=CC=C2OCC=C)=O**
2-amino-7-methyl-5-oxo-4-[4-(trifluoromethoxy)phenyl]-4H,5H-pyrano[4,3-b]pyran-3-carbonitrile	VS#**1**-5	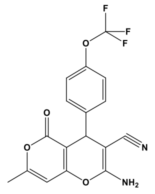 **N#CC(C(C(C=C1)=CC=C1OC(F)(F)F)C(C2=O)=C(C=C(C)O2)O3)=C3N**
[1-(3,4-Difluoro-benzyl)-3-oxo-piperazin-2-yl]-acetic acid	VS#**1**-6	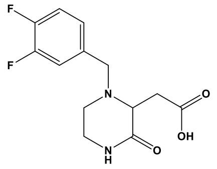 **FC1=CC(CN2C(CC(O)=O)C(NCC2)=O)=CC=C1F**
3-[3-(5-Benzylidene-4-oxo-2-thioxo-thiazolidin-3-yl)-2,5-dioxo-pyrrolidin-1-yl]-propionic acid	VS#**1**-7	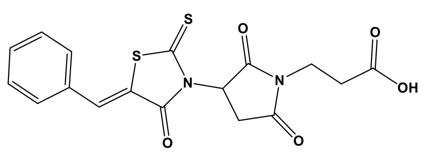 **S=C(S/1)N(C(C1=C\C2=CC=CC=C2)=O)C3C(N(CCC(O)=O)C(C3)=O)=O**
N-cyclopropyl-2-(1,3-dimethyl-2,6-dioxo-1,2,3,6-tetrahydro-9H-purin-9-yl)acetamide	VS#**1**-8	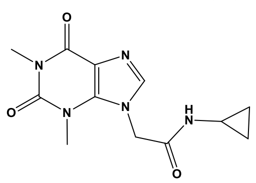 **O=C1N(C)C(C(N=C2)=C(N2CC(NC3CC3)=O)N1C)=O**

6-Amino-5-[2-(4-cyclopropyl-5-pyridin-4-yl-4H-[1,2,4]triazol-3-ylsulfanyl)-acetyl]-1,3-dimethyl-1H-pyrimidine-2,4-dione	VS#**1-**9	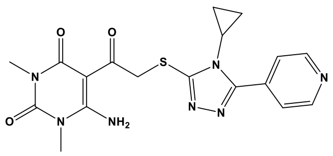<br>**CN(C1=O)C(N)=C(C(N1C)=O)C(CSC(N2C3CC3)=NN=C2C4=CC=NC=C4)=O**
4-(1,3-Dimethyl-2,5-dioxo-1,2,3,6-tetrahydro-purin-7-yl)-butyric acid 1-(3-acetyl-phenylcarbamoyl)-ethyl ester	VS#**1-**10	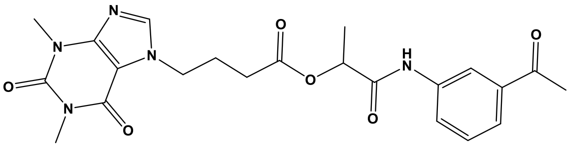<br>**CC(C(NC1=CC(C(C)=O)=CC=C1)=O)OC(CCCN2C=NC(N(C(N3C)=O)C)=C2C3=O)=O**
(1,3-Dimethyl-2,6-dioxo-1,2,3,6-tetrahydro-purin-7-yl)-acetic acid (3-nitro-phenylcarbamoyl)-methyl ester	VS#**1-**11	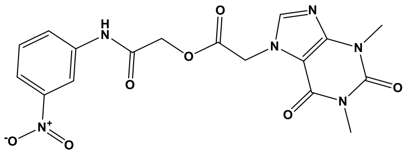<br>**CN(C1=O)C(N(C2=C1N(CC(OCC(NC3=CC([N+]([O-])=O)=CC=C3)=O)=O)C=N2)C)=O**
2-(2-Cyclohexyl-6,8-dimethyl-5,7-dioxo-5,6,7,8-tetrahydro-pyrimido[4,5-d]pyrimidin-4-ylsulfanyl)-N-(5-methyl-isoxazol-3-yl)-acetamide	VS#**1-**12	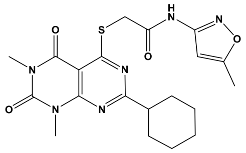<br>**O=C(N1C)N(C)C(N=C(C2CCCCC2)N=C3SCC(NC4=NOC(C)=C4)=O)=C3C1=O**
6-Amino-1,3-dimethyl-5-{2-[2-(piperidine-1-carbonyl)-2,3-dihydro-benzo[1,4]oxazin-4-yl]-acetyl}-1H-pyrimidine-2,4-dione	VS#**1-**13	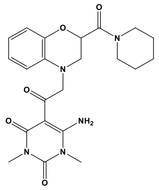<br>**CN(C1=O)C(N)=C(C(N1C)=O)C(CN2CC(C(N3CCCCC3)=O)OC4=C2C=CC=C4)=O**

Table S2B. First virtual screening results (VS#**1**): selection of potential CDA inhibitors according to DF_1.

**Comp. N°**	**GATS4m**	**GGI8**	**DF_1[a]**	**Prob (Active)[b]**	**Inh(%) (exp)[c]**
VS#**1**-1	0.737	0.296	9.7	1.000	67[d]
VS#**1**-2	0.843	0.238	6.6	0.999	57
VS#**1**-3	1.058	0.349	8.3	1.000	54
VS#**1**-4	1.405	0.278	2.2	0.902	38
VS#**1**-5	0.796	0.28	8.6	1.000	35
VS#**1**-6	0.875	0.222	5.7	0.997	28
VS#**1**-7	1.44	0.334	3.8	0.979	23
VS#**1**-8	1.007	0.137	1.4	0.796	16
VS#**1**-9	1.517	0.407	5.6	0.996	0
VS#**1**-10	1.065	0.346	8.1	1.000	0
VS#**1**-11	1.216	0.297	4.8	0.992	0
VS#**1**-12	1.591	0.463	6.8	0.999	0
VS#**1**-13	0.926	0.571	17.4	1.000	0

a Value of the discriminant function, DF_1, obtained for each compound with the Eq. 1
b Probability with which the model classifies it as active
c Experimental inhibition of CDA (%).
d Compounds were tested at the concentration of 1 µM.

Table S3. Calculated logInh(%) activity for all compounds selected in the first virtual screening (VS#1). Experimental results are also reported.

**Comp. Nº**	**Inh(%) (exp)**[a]	**LogInh (exp)**	**T(N..N)**	**JGI2**	**LogInh (cal)**[b]
VS#**1**-1	67	1.826	0	0.102	1.788
VS#**1**-2	57	1.756	0	0.09	1.673
VS#**1**-3	54	1.732	15	0.125	1.732
VS#**1**-4	38	1.58	0	0.082	1.597
VS#**1**-5	35	1.544	4	0.069	1.399
VS#**1**-6	28	1.447	3	0.084	1.561
VS#**1**-7	23	1.362	3	0.078	1.503
VS#**1**-8	16	1.204	30	0.098	1.198
VS#**1**-9	0		122	0.092	-0.552
VS#**1**-10	0		50	0.093	0.782
VS#**1**-11	0		94	0.087	-0.085
VS#**1**-12	0		72	0.077	0.225
VS#**1**-13	0		50	0.092	0.773

[a] experimental inhibitory value (Log)
[b] calculated inhibitory value (Log) with Eq. 2

Table S4A. Second selection of potential CDA inhibitors (virtual screening number 2, VS#**2**) applying equations 1 and 2.

Name	Comp Nº	Inh% (exp)	Srtucture and SMILES code
{3-[(1,3-dimethyl-2,4,6-trioxotetrahydro-5(2H)-pyrimidinylidene)methyl]phenoxy}acetic acid	VS#**2**-1	80	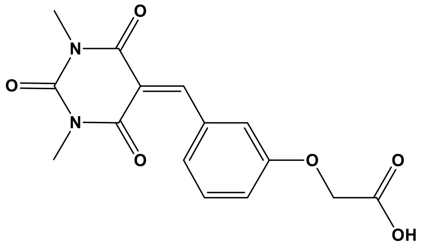<br>**O=C(N(C)C/1=O)N(C)C(C1=C\C2=CC=CC(OCC(O)=O)=C2)=O**
(1-methyl-2,4-dioxo-1,4,6,7-tetrahydro[1,3]thiazole[2,3-f]purin-3(2H)-yl)acetic acid	VS#**2**-2	63	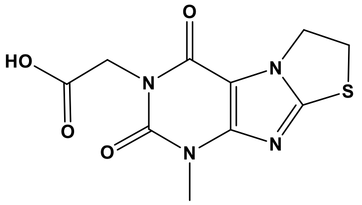<br>**O=C(N(C)C(N=C1SCCN21)=C2C3=O)N3CC(O)=O**
2-(2-methoxy-4-{[(5Z)-2,4,6-trioxo-1-(prop-2-en-1-yl)-1,3-diazinan-5-ylidene]methyl}phenoxy)acetic acid	VS#**2**-3	64	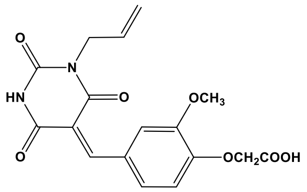<br>**O=C(NC/1=O)N(CC=C)C(C1=C/C2=CC=C(C(OC)=C2)OCC(O)=O)=O**
[2-(1,3-Dimethyl-4,6-dioxo-2-thioxo-tetrahydro-pyrimidin-5-ylidenemethyl)-phenoxy]-acetic acid	VS#**2**-4	38	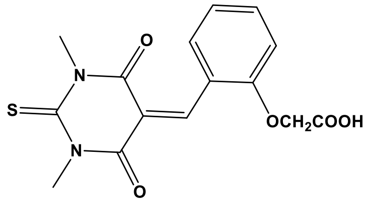<br>**S=C(N(C)C/1=O)N(C)C(C1=C/C2=CC=CC=C2OCC(O)=O)=O**
[4,6-Dioxo-7-(thiazol-2-ylcarbamoylmethyl)-2,3,6,7-terahydro-1H,4H-8-thia-5,7-diaza-cyclopentana[a]inden-5-yl]-acetic acid	VS#**2**-5	37	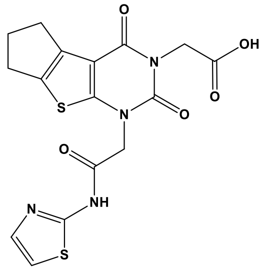<br>**O=C(N(CC(NC1=NC=CS1)=O)C2=C3C(CCC4)=C4S2)N(CC(O)=O)C3=O**
3-(1H-Imidazol-4-yl)-2-{[1-(4-isopropyl-phenyl)-2-mercapto-4,6-dioxo-1,6-dihydro-4H-pyrimidin-5-ylidenemethyl]-amino}-propionic acid	VS#**2**-6	28	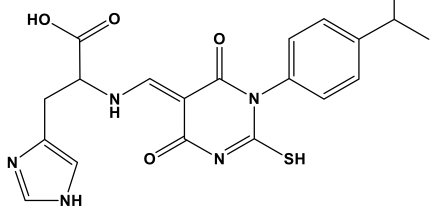<br>**O=C(/C1=C/NC(CC2=CNC=N2)C(O)=O)N(C3=CC=C(C(C)C)C=C3)C(S)=NC1=O**

N-{4-[3-(2,6-dimethyl-4-morpholinyl)-2,5-dioxo-1-pyrrolidinyl]phenyl}acetamide	VS#**2**-7	26	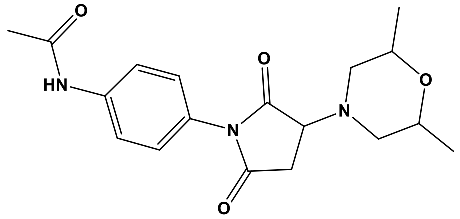 **O=C(C1N2CC(OC(C)C2)C)N(C(C=C3)=CC=C3NC(C)=O)C(C1)=O**
2-[4-(1,3-Dimethyl-2,4,6-trioxo-tetrahydro-pyrimidin-5-ylidenemethyl)-2-methoxy-phenoxy]-propionic acid	VS#**2**-8	26	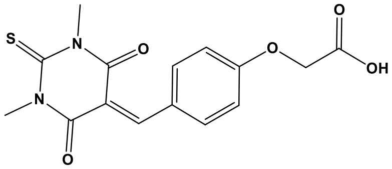 **S=C(N(C)C/1=O)N(C)C(C1=C/C(C=C2)=CC=C2OCC(O)=O)=O**
2-(1,3-dimethyl-2,6-dioxo-1,2,3,6-tetrahydro-7H-purin-7-yl)-N-(2-methoxy-1-methylethyl)acetamide	VS#**2**-9	25	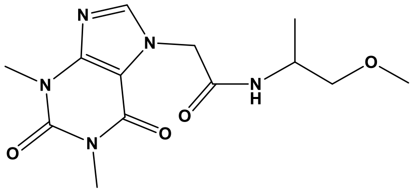 **O=C(N(C)C(N=CN1CC(NC(C)COC)=O)=C1C2=O)N2C**
2-({5-[Methyl-(2-methyl-4-oxo-1,4-dihydro-quinazolin-6-ylmethyl)-amino]-thiophene-2-carbonyl}-amino)-pentanedioic acid	VS#**2**-10	20	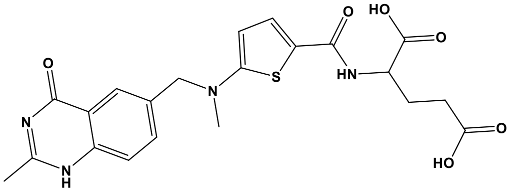 **CC(NC1=CC=C(CN(C)C2=CC=C(C(NC(CCC(O)=O)C(O)=O)=O)S2)C=C31)=NC3=O**
3-benzyl-1,7-dimethyl-7,9-dihydro-1H-purine-2,6,8(3H)-trione	VS#**2**-11	13	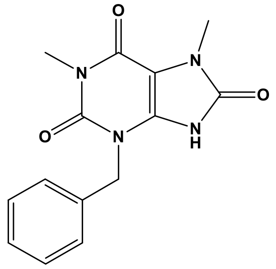 **O=C(N(CC1=CC=CC=C1)C(NC2=O)=C(N2C)C3=O)N3C**
[3-(2-Chloro-6-fluoro-benzyl)-2,4,5-trioxo-imidazolin-1-yl]-acetic acid	VS#**2**-12	9	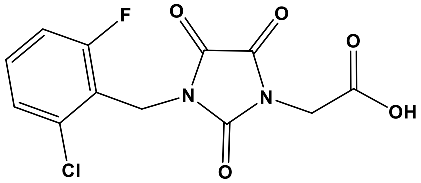 **O=C(N(CC(C(Cl)=CC=C1)=C1F)C2=O)N(CC(O)=O)C2=O**
N-(3-Chloro-2-methyl-phenyl)-2-[3-oxo-1-(2,2,2-trifluoro-acetyl)-piperazin-2-yl]-acetamide	VS#**2**-13	3	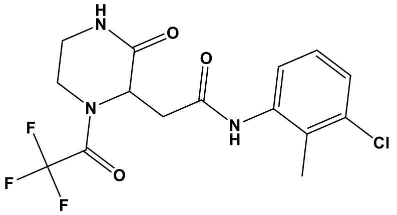 **O=C(C(F)(F)F)N1C(CC(NC2=CC=CC(Cl)=C2C)=O)C(NCC1)=O**

5-(2-Hydroxy-5-nitro-benzylidene)-1,3-dimethyl-pyrimidine-2,4,6-trione	VS#**2**-14	0	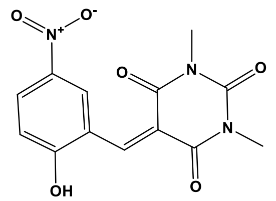<br>**O=C(N(C)C/1=O)N(C)C(C1=C\C2=CC([N+]([O-])=O)=CC=C2O)=O**
[4-(1,3-Dimethyl-4,6-dioxo-2-thioxo-tetrahydro-pyrimidin-5-ylidenemethyl)-phenoxy]-acetic acid	VS#**2**-15	0	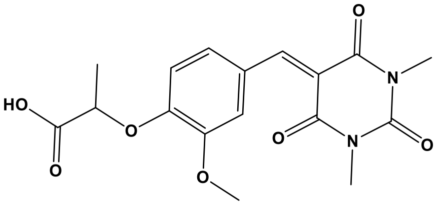<br>**O=C(N(C)C/1=O)N(C)C(C1=C/C2=CC(OC)=C(C=C2)OC(C)C(O)=O)=O**
3-(1,3-Dioxo-octahydro-isoindol-2-yl)-propionic acid 2-(6-amino-1,3-dimethyl-2,4-dioxo-1,2,3,4-tetrahydro-pyrimidin-5-yl)-2-oxo-ethyl ester	VS#**2**-16	0	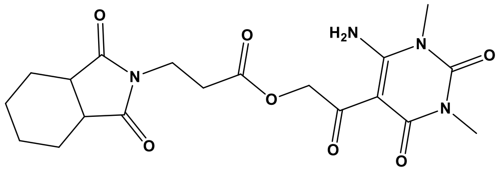<br>**CN(C1=O)C(N)=C(C(N1C)=O)C(COC(CCN2C(C3CCCCC3C2=O)=O)=O)=O**
Butyric 2-(6-amino-1-benzyl-3-methoxycarbonylmethyl-2,4-dioxo-1,2,3,4-tetrahydro-pyrimidin-5-yl)-2-oxo-ethyl ester	VS#**2**-17	0	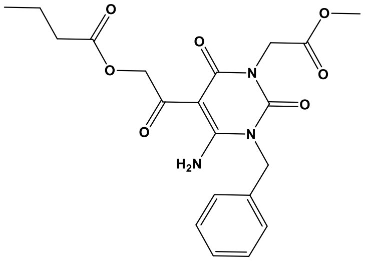<br>**CCCC(OCC(C(C1=O)=C(N(C(N1CC(OC)=O)=O)CC2=CC=CC=C2)N)=O)=O**
N-(2-Chloro-5-trifluoromethyl-phenyl)-2-(3-nitro-[1,2,4]triazol-1-yl)-acetamide	VS#**2**-18	0	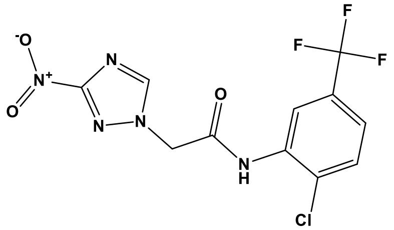<br>**[O-][N+](C(N=C1)=NN1CC(NC2=CC(C(F)(F)F)=CC=C2Cl)=O)=O**
3-Methyl-thiophene-2-carboxylic acid 2-(6-amino-1-benzyl-3-methoxycarbonylmethyl-2,4-dioxo-1,2,3,4-tetrahydro-pyrimidin-5-yl)-2-oxo-ethyl ester	VS#**2**-19	0	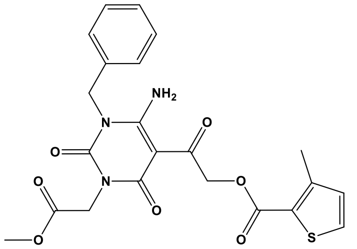<br>**COC(CN(C1=O)C(C(C(COC(C2=C(C)C=CS2)=O)=O)=C(N1CC3=CC=CC=C3)N)=O)=O**
6-Amino-5-[2-(4-isopropyl-5-oxo-1-p-totyl-4,5-dihydro-1H-imidazol-2-yl sulfanyl)-acetyl]-3-methyl-1-propyl-1H-pyrimidine-2,4-dione	VS#**2**-20	0	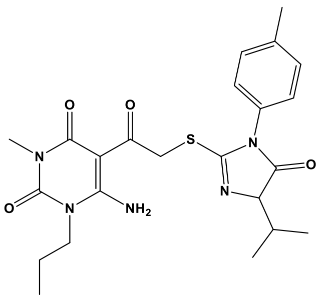<br>**CCCN(C1=O)C(N)=C(C(N1C)=O)C(CSC(N2C3=CC=C(C)C=C3)=NC(C2=O)C(C)C)=O**

Table S4B. List of the compounds chosen after the second virtual screening (VS#**2**) is reported. The value of DF_1 and Log(Inh%)Calc, as well as the values of the topological indices for each molecule are reported.

**CompNº**	**%Inh (exp)**	**GATS4m**	**GGl8**	**DF_1**	**P.A.**	**T(N..N)**	**JGl2**	**LogInh cal**	**%Inh (calc)**
VS#**2**-1	80.00	0.65	0.23	8.44	1.00	0.00	0.09	1.69	49.00
VS#**2**-2	63.00	0.70	0.06	1.96	0.88	10.00	0.08	1.42	26.00
VS#**2**-3	64.00	0.97	0.24	5.27	0.99	0.00	0.09	1.67	46.77
VS#**2**-4	38.00	0.87	0.28	7.87	1.00	0.00	0.11	1.82	66.00
VS#**2**-5	37.00	0.83	0.34	10.36	1.00	20.00	0.07	1.08	12.00
VS#**2**-6	28.00	1.41	0.47	8.80	1.00	51.00	0.09	0.73	5.00
VS#**2**-7	26.00	0.91	0.33	9.07	1.00	16.00	0.07	1.17	15.00
VS#**2**-8	26.00	0.92	0.19	4.12	0.98	0.00	0.10	1.75	56.00
VS#**2**-9	25.00	1.07	0.22	3.80	0.98	30.00	0.09	1.16	14.00
VS#**2**-10	20.00	0.83	0.33	10.16	1.00	39.00	0.08	0.89	8.00
VS#**2**-11	13.00	0.95	0.12	1.47	0.81	10.00	0.09	1.44	28.00
VS#**2**-12	9.00	0.97	0.20	4.02	0.98	0.00	0.10	1.81	64.00
VS#**2**-13	3.00	0.81	0.34	10.55	1.00	12.00	0.11	1.60	39.00
VS#**2**-14	0.00	0.85	0.25	6.83	1.00	14.00	0.12	1.68	48.00
VS#**2**-15	0.00	0.72	0.30	9.92	1.00	0.00	0.11	1.85	70.00
VS#**2**-16	0.00	0.91	0.30	7.95	1.00	31.00	0.11	1.29	19.00
VS#**2**-17	0.00	1.04	0.37	9.36	1.00	4.00	0.09	1.55	36.00
VS#**2**-18	0.00	1.00	0.28	6.33	1.00	21.00	0.09	1.28	19.00
VS#**2**-19	0.00	0.52	0.47	18.16	1.00	4.00	0.09	1.61	41.00
VS#**2**-20	0.00	1.62	0.61	11.72	1.00	46.00	0.10	0.92	8.00

Table S5A. Descriptors values, DF_2 value for training set compounds, probability of being classified as potential CDA inhibitor, and classification as active or inactive by the model. Quantitative and qualitative experimental values related to fungicide activity is also reported.

**Comp. Nº**	**Inh% (exp)[a]**	**Clas (exp)[b]**	**GGI10**	**SEige**	**GATS3e**	**DF_2[c]**	**Prob (Act)[d]**	**Clas (calc)[d]**
**Active group**								
VS#**2**-1	80	A	0.112	1.755	1.052	1.6	0.828	A
VS#**1**-1	67	A	0.103	1.755	1.099	1.4	0.798	A
VS#**2**-2	63	A	0	1.609	0.936	5.4	0.996	A
VS#**2**-3	64	A	0.165	2.002	0.935	0.2	0.551	A
VS#**1**-2	57	A	0.165	2.002	0.935	0.2	0.551	A
VS#**1**-3	54	A	0.049	2.788	1.103	-2.9	0.051	I
VS#**2**-4	38	A	0.058	1.58	0.95	4.3	0.987	A
VS#**1**-4	38	A	0.144	1.759	0.917	2.0	0.880	A
VS#**2**-5	37	A	0.081	1.927	0.939	2.2	0.899	A
VS#**1**-5	35	A	0.099	2.2	0.725	2.1	0.895	A
VS#**2**-6	28	A	0.133	1.747	1.029	1.4	0.800	A
VS#**1**-6	28	A	0	1.641	0.838	6.0	0.998	A
VS#**2**-7	26	A	0.116	1.4	0.803	5.3	0.995	A
VS#**2**-8	26	A	0.136	1.58	0.899	3.2	0.961	A
VS#**2**-9	25	A	0.07	1.676	1.053	2.8	0.942	A
VS#**1**-7	23	A	0.057	1.651	1.006	3.5	0.972	A
VS#**2**-10	20	A	0.244	2.102	0.848	-1.2	0.241	I
VS#**1**-8	16	A	0.02	1.429	1.158	4.2	0.985	A
VS#**2**-11	13	A	0	1.292	1.25	4.6	0.989	A
VS#**2**-12	9	A	0.025	2.031	1.07	1.7	0.846	A
**Inactive group**								
VS#**2**-13	3	I	0.136	2.301	0.571	2.1	0.893	A
VS#**1**-9	0	I	0.259	1.776	1.338	-3.7	0.025	I
VS#**2**-14	0	I	0	1.893	1.346	0.7	0.671	A
VS#**1**-10	0	I	0.222	2.169	1.038	-2.6	0.071	I
VS#**1**-11	0	I	0.16	2.554	0.987	-2.9	0.051	I
VS#**1**-12	0	I	0.23	1.885	1.137	-2.1	0.113	I
VS#**2**-15	0	I	0.178	2.002	1.073	-1.1	0.243	I
VS#**1**-13	0	I	0.252	1.923	1.154	-2.8	0.057	I
VS#**2**-20	0	I	0.362	1.747	1.294	-5.2	0.006	I
VS#**2**-16	0	I	0.231	2.278	1.276	-5.2	0.006	I
VS#**2**-19	0	I	0.243	2.211	1.061	-3.4	0.033	I
VS#**2**-17	0	I	0.165	2.14	1.043	-1.4	0.205	I
VS#**2**-18	0	I	0.147	2.577	0.958	-2.6	0.072	I

[a] Fungal growth inhibitory % for each compound based on experimental assays;
[b] Classification of the compounds based on experimental assays;
[c] Value of the discriminant function, DF_2 from Eq. 3
[d] Probability of activity and classification based on DF_2 results.

Table S5B. LSO internal validation procedure for DF_2 (1=training active group; 2=training inactive group; 3=test active group; 4=test inactive group).

**Comp. Nº**	**Inh% (exp)**	**Class. DF2**	**P.A. DF2**	**Class. LSO1**	**P.A. LSO1**	**Class. LSO2**	**P.A. LSO2**	**Class. LSO3**	**P.A. LSO3**	**Class. LSO4**	**P.A. LSO4**
VS#**2**-1	80	1	0.867	1	0.901	1	0.952	1	0.814	3	0.8573
VS#**1**-1	67	1	0.843	1	0.866	1	0.964	3	0.785	1	0.7485
VS#**2**-2	63	1	0.997	1	0.998	3	1.000	1	0.990	1	0.9996
VS#**1**-2	57	1	0.645	3	0.618	1	0.416	1	0.618	1	0.9136
VS#**2**-4	38	1	0.990	1	0.996	1	0.998	1	0.975	3	0.9967
VS#**1**-4	38	1	0.906	1	0.956	1	0.865	3	0.867	1	0.9739
VS#**2**-5	37	1	0.933	1	0.907	3	0.975	1	0.885	1	0.9926
VS#**1**-5	35	1	0.939	3	0.858	1	0.874	1	0.895	1	0.9998
VS#**2**-6	28	1	0.840	1	0.901	1	0.897	1	0.792	3	0.8379
VS#**1**-6	28	1	0.998	1	0.999	1	1.000	3	0.994	1	0.9999
VS#**2**-7	26	1	0.995	1	1.000	3	0.994	1	0.989	1	0.9993
VS#**2**-8	26	1	0.967	3	0.993	1	0.959	1	0.944	1	0.9881
VS#**2**-9	25	1	0.956	1	0.970	1	0.995	1	0.919	3	0.9579
VS#**1**-7	23	1	0.979	1	0.987	1	0.998	3	0.954	1	0.9892
VS#**2**-10	20	1	0.312	1	0.345	3	0.016	1	0.368	1	0.8412
VS#**1**-8	16	1	0.988	3	0.995	1	1.000	1	0.969	1	0.9381
VS#**2**-11	13	1	0.991	1	0.997	1	1.000	1	0.975	3	0.8261
VS#**2**-12	9	1	0.907	1	0.702	1	0.995	3	0.834	1	0.9774
VS#**2**-3	64	1	0.645	1	0.618	3	0.416	1	0.618	1	0.9136
VS#**1**-3	54	1	0.130	3	0.002	1	0.575	1	0.126	1	0.8273
VS#**2**-13	3	2	0.939	2	0.874	2	0.535	2	0.902	4	1.0000
VS#**1**-9	0	2	0.029	2	0.051	2	0.017	4	0.057	2	0.0002
VS#**2**-14	0	2	0.771	2	0.412	4	0.999	2	0.663	2	0.2577
VS#**1**-10	0	2	0.104	4	0.056	2	0.020	2	0.149	2	0.1575
VS#**1**-11	0	2	0.099	2	0.008	2	0.045	2	0.124	4	0.7019
VS#**1**-12	0	2	0.140	2	0.190	2	0.049	4	0.194	2	0.0247
VS#**2**-15	0	2	0.317	2	0.252	4	0.234	2	0.343	2	0.2897
VS#**1**-13	0	2	0.071	4	0.094	2	0.015	2	0.116	2	0.0087
VS#**2**-20	0	2	0.006	2	0.024	2	0.000	2	0.018	4	0.0000
VS#**2**-16	0	2	0.009	2	0.002	2	0.005	4	0.020	2	0.0009
VS#**2**-19	0	2	0.050	2	0.024	4	0.006	2	0.084	2	0.0556
VS#**2**-17	0	2	0.292	4	0.141	2	0.219	2	0.313	2	0.4937
VS#**2**-18	0	2	0.140	2	0.011	2	0.072	2	0.162	4	0.8614

Red colour: misclassified compounds by the model.

Table S6A. Third selection of potential CDA inhibitors (virtual screening number 3, VS#**3**).

Name	Comp. Nº	Srtucture and SMILES code
2-({[(5E)-4,6-dioxo-1-[4-(propan-2-yl)phenyl]-2-sulfanyl-1,4,5,6-tetrahydropyrimidin-5-ylidene]methyl}amino)-3-(1H-imidazol-4-yl)propanoic acid	VS#**3**-1	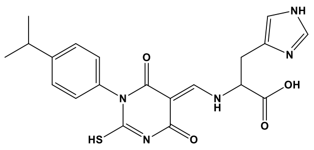<br>**O=C(O)C(N/C=C1C(N=C(S)N(C2=CC=C(C(C)C)C=C2)C\1=O)=O)CC3=CNC=N3**
2-(10,12-dioxo-9-{[(1,3-thiazol-2-yl)carbamoyl]methyl}-7-thia-9,11-diazatricyclo[6.4.0.$0^{2,6}$]dodeca-1(8),2(6)-dien-11-yl)acetic acid	VS#**3**-2	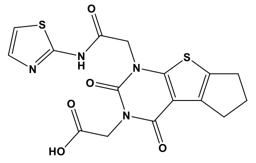<br>**O=C(O)CN(C1=O)C(N(CC(NC2=NC=CS2)=O)C3=C1C4=C(S3)CCC4)=O**
2-[(5E)-4-oxo-5-{[2-(prop-2-en-1-yloxy)phenyl]methylidene}-2-sulfanylidene-1,3-thiazolidin-3-yl]pentanedioic acid	VS#**3**-3	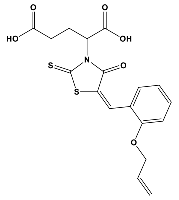<br>**O=C(O)C(N(C/1=O)C(SC1=C\C2=CC=CC=C2OCC=C)=S)CCC(O)=O**
2-{1-[(3,4-difluorophenyl)methyl]-3-oxopiperazin-2-yl}acetic acid	VS#**3**-4	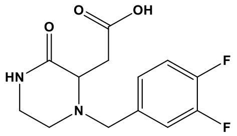<br>**O=C(O)CC1N(CC2=CC=C(F)C(F)=C2)CCNC1=O**
2-{3-[(2-chloro-6-fluorophenyl)methyl]-2,4,5-trioxoimidazolidin-1-yl}acetic acid	VS#**3**-5	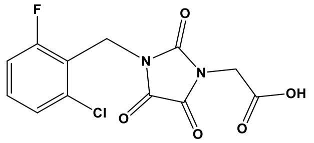<br>**O=C(O)CN(C(N(CC1=C(F)C=CC=C1Cl)C2=O)=O)C2=O**
2-{4-[(1,3-dimethyl-4,6-dioxo-2-sulfanylidene-1,3-diazinan-5-ylidene)methyl]phenoxy}acetic acid	VS#**3**-6	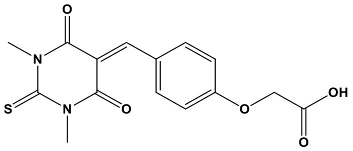<br>**O=C(O)COC1=CC=C(/C=C2C(N(C)C(N(C)C\2=O)=S)=O)C=C1**
3-{2,5-dioxo-3-[(5Z)-4-oxo-5-(phenylmethylidene)-2-sulfanylidene-1,3-thiazolidin-3-yl]pyrrolidin-1-yl}propanoic acid,	VS#**3**-7	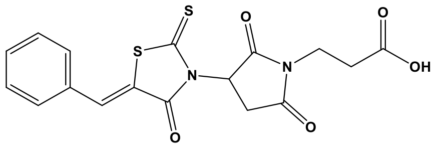<br>**O=C(O)CCN1C(C(N(C/2=O)C(SC2=C/C3=CC=CC=C3)=S)CC1=O)=O**
5-imino-1-(2-methyl-5-nitrophenyl)-3-phenylhydantoin,	VS#**3**-8	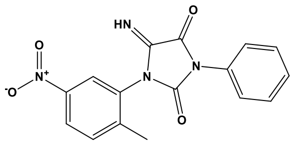<br>**O=C(N(C1=CC=CC=C1)C2=O)N(C3=CC([N+]([O-])=O)=CC=C3C)C2=N**

5-imino-1-(4-methyl-5-nitrophenyl)-3-phenylhydantoin,	VS#**3**-9	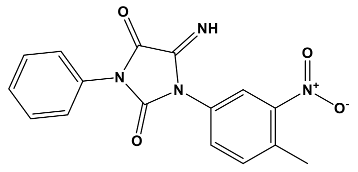<br>**O=C(N(C1=CC=CC=C1)C2=O)N(C3=CC([N+]([O-])=O)=C(C)C=C3)C2=N**
7-[2-hydroxy-3-(4-morpholinyl)propyl]-1,3-dimethyl-3,7-dihydro-1H-purine-2,6-dione,	VS#**3**-10	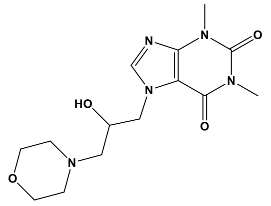<br>**O=C(N1C)N(C)C2=C(N(CC(O)CN3CCOCC3)C=N2)C1=O**

Table S6B. Virtual screening number 3, applying DF_1, logInh and DF_2 (Eq.1, Eq.2 and Eq.3). Descriptors and equations values are listed.

Comp. Nº	G4m	GI8	DF_1	TN	JI2	logI	GI10	Sei	G3e	DF_2
VS#**3**-1	1.41	0.47	8.80	51	0.09	0.70	0.13	1.75	1.03	1.40
VS#**3**-2	0.83	0.34	10.36	20	0.07	1.10	0.08	1.93	0.94	2.90
VS#**3**-3	1.41	0.28	2.22	0	0.08	1.60	0.14	1.76	0.92	2.00
VS#**3**-4	0.88	0.22	5.71	3	0.08	1.60	0.00	1.64	0.84	6.00
VS#**3**-5	0.97	0.20	4.02	0	0.10	1.80	0.03	2.03	1.07	1.70
VS#**3**-6	0.92	0.19	4.12	0	0.10	1.80	0.14	1.58	0.90	3.20
VS#**3**-7	1.44	0.33	3.82	3	0.08	1.50	0.06	1.65	1.01	3.60
VS#**3**-8	1.15	0.21	2.59	22	0.09	1.30	0.06	1.54	1.04	3.90
VS#**3**-9	1.17	0.15	0.17	16	0.08	1.30	0.05	1.54	1.02	4.20
VS#**3**-10	1.00	0.22	4.37	34	0.08	1.00	0.10	1.68	0.91	3.40

G4m=GATS4m;G3e=GATS3e;GI8= GGI8;GI10=GGI10;TN=T(N..N);JI2=JGI2;logI=log Inh cal; Sei=SEige.

Table S7A. Fungicidal effect of the compounds identified by molecular topology approach on cucurbit powdery mildew development (*P. xanthii*) in the leaf disc assay.

Compound	Concentration (µM)	*Podosphaera xanthii*			
		Strain 2086		Strain SF60	
		Mean[a]	Efficacy[b] (%)	Mean[a]	Efficacy[b] (%)
Water	-	13.47	-	29.3	-
Acetone (1%)	-	12.90	4.26	27.35	6.65
EDTA (20 mM)	-	0	100	2.54	91.33
Fluopyram	10	0	100	0	100
	100	0	100	0	100
VS#**2-2**	1	4.77	64.58	10.99	62.5
	100	4.54	66.28	14.01	52.17
VS#**2-1**	1	10.18	24.43	5.78	**80.29**
	100	11.79	12.5	16.92	42.25
VS#**1**-1	1	12.16	9.75	9.44	67.79
	100	12.30	8.71	5.65	80.7
VS#**3**-10	1	13.47	0.00	26.71	8.83
	100	13.7	0.00	29.30	0.00
VS#**1**-5	1	11.29	16.19	18.99	35.19
	100	12.22	9.28	27.11	7.47
VS#**2**-7	1	12.14	9.84	22.12	24.49
	100	10.38	22.91	23.97	18.2
VS#**2**-9	1	11.45	14.96	21.74	25.81
	100	13.00	3.50	25.96	11.41
VS#**1**-8	1	10.15	24.62	24.40	16.71
	100	12.46	7.48	21.18	27.71
VS#**2**-11	1	9.85	26.89	25.44	13.17
	100	12.08	10.32	28.90	1.35
VS#**3-8**	1	13.37	0.75	26.00	11.27
	100	13.47	0.00	22.93	21.73
VS#**3-9**	1	13.37	0.75	29.10	0.67
	100	13.47	0.00	29.30	0.00
VS#**3-7**	1	9.64	28.43	18.96	35.29
	100	4.49	66.66	20.84	28.87
VS#**3-2**	1	10.21	24.20	15.59	46.79
	100	9.09	32.51	27.34	6.68
VS#**3-6**	1	6.70	50.25	24.97	14.77
	100	10.41	22.71	18.23	37.78
VS#**3-1**	1	5.13	61.91	17.86	39.04
	100	10.08	25.16	19.53	33.34
VS#**2**-3	1	6.50	51.74	10.54	64.02
	100	12.30	8.68	21.31	27.26
VS#**3-3**	1	5.03	62.65	15.37	47.54
	100	11.70	13.14	29.79	0.00
VS#**3-4**	1	9.99	25.83	17.68	39.65
	100	11.33	15.88	31.38	0.00
VS#**3-5**	1	10.73	20.40	22.43	23.44
	100	10.42	22.64	24.17	17.50

[a] The values represent the average diameter of the fungal colonies expressed in mm.
[b] The values represent the percentage of efficacy of the compounds according to Abbott's formula.
Red color, best fungicide candidates identified by MT.

Table S7B. Fungicidal effect of the compounds identified by molecular topology approach on the cucurbit powdery mildew *P. xanthii* in the melon seedling assay.

**Compound**[a]	**Mean**[b]	**Efficacy**[d]
Water	79.44 a[c]	-
Acetone (1%)	79.06 ab	0.48
EDTA (20 mM)	7.38 f	90.71
Fluopyram	0 g	100
VS#**2**-2	13.42 e	83.11
VS#**2**-1	3.79 f	95.22
VS#**1**-1	61.95 d	22.02
VS#**3**-10	58.71 d	26.10
VS#**1**-5	78.61 ab	1.05
VS#**2**-7	75.89 ab	4.48
VS#**2**-9	77.83 ab	2.03
VS#**1**-8	79.25 ab	0.24
VS#**2**-11	78.37 ab	1.35
VS#**3**-8	80.33 ab	0.00
VS#**3**-9	77.90 ab	1.94
VS#**3**-7	38.92 c	51.01
VS#**3**-2	76.93 ab	3.17
VS#**3**-6	76.18 ab	4.11
VS#**3**-1	77.64 ab	2.27
VS#**2**-3	5.75 f	92.76
VS#**3**-3	74.74 ab	5.93
VS#**3**-4	72.93 b	8.20
VS#**3**-5	72.96 abc	8.17

[a] The compounds and the fungicide fluopyram were applied using solutions at 100 µM.
[b] The values represent the average percentage of leaf area covered by fungal growth.
[c] Different letters are significantly different at *P*=0.05 according to Fisher's least significant difference test (LSD).
[d] The values represent the percentage of efficacy of the compounds according to Abbott's formula.
Red color, best fungicide candidates identified by MT.

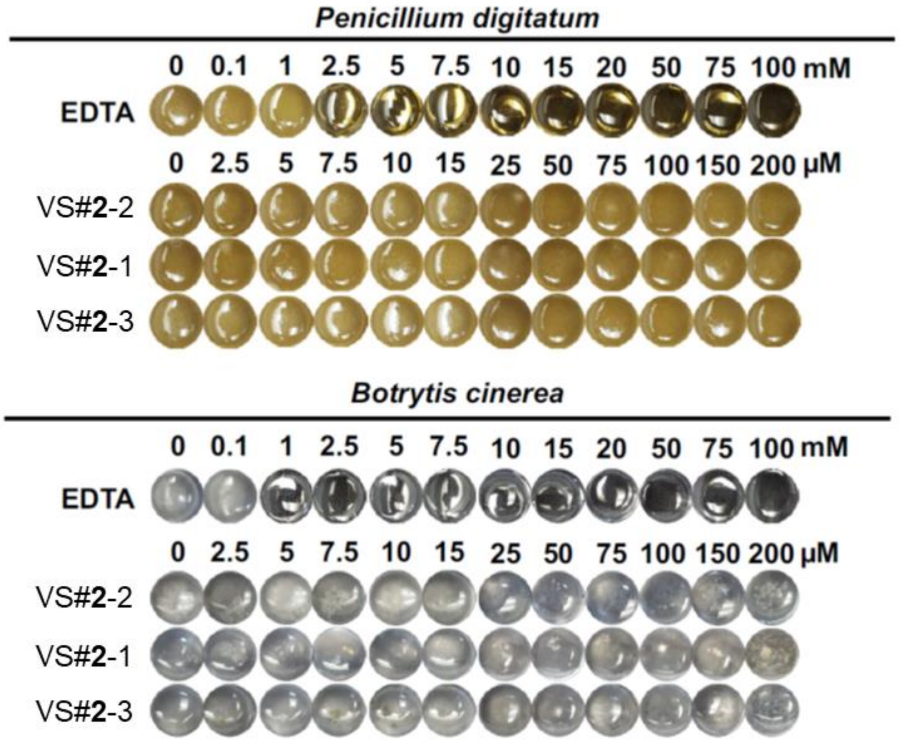

Figure S1. *In vitro* antifungal activity of the compounds identified by molecular topology**.** A microplate assay was used to test the toxicity of the identified compounds on *P. digitatum* and *B. cinerea*. No toxicity was observed for the three compounds in the range of concentrations tested (2.5-200 µM). EDTA was toxic in the range 1 to 2.5 mM.
